# Crosslinking strategies govern the morphology and biological performance of decellularized extracellular matrix particle–tyramine-modified hyaluronic acid hydrogels

**DOI:** 10.1039/d6ra01580h

**Published:** 2026-08-24

**Authors:** Jiangyao Xu, Jacek K. Wychowaniec, Matteo D'Este, Nan Jiang, Songsong Zhu, Sibylle Grad, Jeroen Geurts, Zhen Li

**Affiliations:** a AO Research Institute Davos Davos Platz Switzerland zhen.li@aofoundation.org; b Lausanne University Hospital Lausanne Switzerland; c State Key Laboratory of Oral Diseases, National Clinical Research Center for Oral Diseases, West China Hospital of Stomatology, Sichuan University Chengdu China; d SDU Biotechnology, Department of Green Technology, Faculty of Engineering, University of Southern Denmark Campusvej 55 5230 Odense Denmark

## Abstract

Decellularized extracellular matrix (dECM) particle-based hydrogels hold promise for cartilage repair. Due to its chondropermissive properties, tuneable viscoelastic profile, and the potential to form covalent bonds with ECM proteins, tyramine-modified hyaluronic acid (THA) is an ideal candidate as a binder for dECM particles. However, the incorporation of the dECM particles into a viscoelastic hydrogel may interfere with its crosslinking. The goal of this study was to compare two crosslinking methods: (1) horseradish peroxidase/Eosin (HRP/Eo) with H_2_O_2_ and (2) sodium persulfate/ruthenium (SPS/Ru) for fabricating THA hydrogels containing dECM particles, and to investigate their biological and biomechanical effect for cartilage tissue engineering. Incorporation of dECM markedly enhanced the mechanical properties of the composites in both crosslinking methods, increasing the storage modulus by 10-fold, while leaving the linear viscoelastic region unchanged. Chondrocyte-laden dECM hydrogels demonstrated sustained cytocompatibility over 14 days, evidenced by stable DNA content throughout the culture period. Compared with SPS/Ru, hydrogels showed more effective incorporation of dECM particles into the HRP/Eo-crosslinked network, as indicated by a lower *I*_∼1630_/*I*_∼1030_ ratio from FTIR analysis, clear morphological changes in confocal microscopy, higher glycosaminoglycan (GAG) and collagen retention, as well as slower GAG and collagen release. HRP/Eo-dECM hydrogels promoted stronger gene expression of type II collagen (COL2) and higher compressive modulus compared with SPS/Ru-dECM hydrogels. In summary, HRP/Eo crosslinked dECM hydrogels demonstrated improved mechanical performance and chondrocyte redifferentiation effect compared to SPS/Ru, attributed to the difference in hydrogel morphology. These findings identify the HRP/Eo crosslinking method as a favorable strategy for engineering dECM particle–THA hydrogels for cartilage regeneration.

## Abstract 2

Decellularized extracellular matrix (dECM) particle-based hydrogels hold promise for cartilage repair.

## Graphical abstract

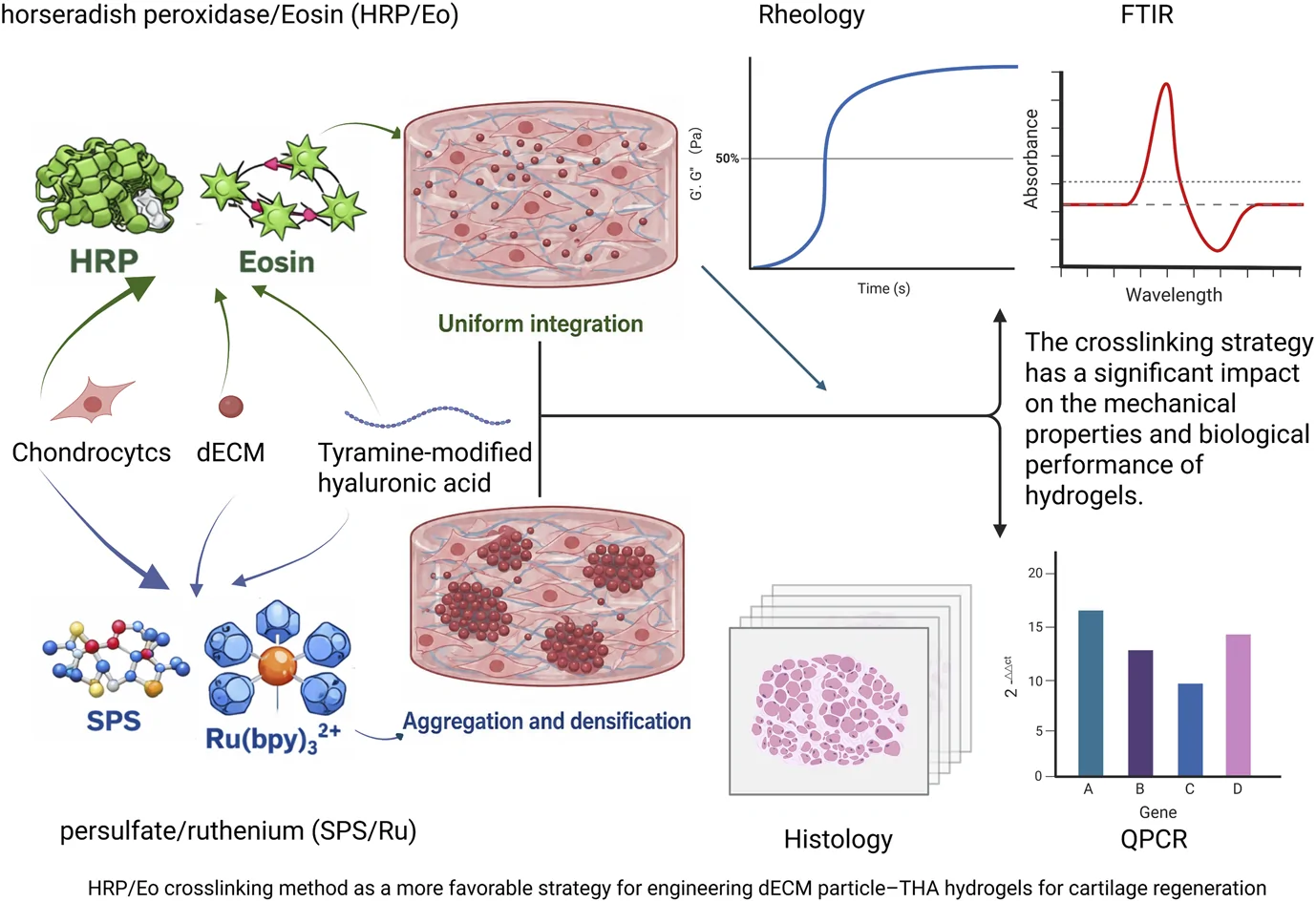

## 1. Introduction

Multi-component hydrogels that integrate bioactive extracellular matrix (ECM) components with mechanically tunable polymer networks have gained increasing attention for cartilage repair, as they enable the decoupling and optimization of biological and mechanical functions within a single material system.^1^ In such composite hydrogels, the choice of crosslinking chemistry plays a critical role in governing gelation kinetics, network architecture, mechanical stability, and cellular responses.^2,3^ However, systematic investigations into how different crosslinking strategies influence the functional performance of ECM-containing hydrogels remain limited.^4^ Addressing this knowledge gap is essential for the rational design of mechanically robust yet biologically supportive hydrogel systems for cartilage regeneration.^5^

Cartilage is an avascular and aneural tissue with limited self-repair capacity, making it challenging to restore its structure and function after damage.^6,7^ As a result, effective cartilage repair remains a major clinical challenge.^8^ In recent years, hydrogels have emerged as promising biomaterials for cartilage tissue engineering due to their excellent biocompatibility, tunable mechanical properties, and three-dimensional structure, which closely resemble cartilage tissue.^9^ Hydrogels not only provide a matrix-like microenvironment for cells but also support cell proliferation, differentiation, and new matrix generation.^9–12^ Furthermore, hydrogels can fill irregularly shaped cartilage defects *via* minimally invasive procedures, further broadening their clinical applications.^13,14^ Notably, incorporating dECM into THA–based hydrogels enriches the hydrogels with bioactive signals, promoting the functional recovery of chondrocytes.^15–18^ dECM-embedded hydrogels have been shown to enhance chondrocyte proliferation, maintain chondrogenic phenotype, and promote the synthesis of cartilage-specific extracellular matrix components, thereby supporting functional tissue regeneration.^6,7,16^ The integration of dECM not only improves the biological activity of hydrogels but can also influence mechanical properties, swelling behavior, and degradation kinetics, making it a versatile strategy for cartilage tissue engineering.^8,19^ To this end, numerous composite hydrogels containing dECM particles have been developed for cartilage tissue engineering or repair.^20–23^

Hyaluronic acid (HA) is one of the main components of the native cartilage ECM. It has been widely used as a starting material for hydrogel fabrication due to its inherent biocompatibility and hydrophilicity.^7^ HA-based hydrogels provide a favorable environment for chondrocyte survival and cartilage-specific matrix deposition.^24–28^ Chemical modification of HA with functional groups, such as tyramine, allows enzymatic or photochemical crosslinking, resulting in hydrogels with tunable mechanical strength, gelation kinetics, and degradation profiles.^29,30^ Tyramine-modified hyaluronic acid (THA) hydrogels have been extensively applied in cartilage repair, either alone or in combination with bioactive components, including dECM particles, demonstrating improved chondrogenesis and tissue integration.^31^ The immunomodulatory function of THA has also been confirmed and shown to depend on the molecular weight of the polymer used,^32^ paving the way for their future translational use.

Horseradish peroxidase/Eosin (HRP/Eo^33,34^) and persulfate/ruthenium (SPS/Ru^35^) are two viable methods for crosslinking THA with distinct features. The HRP/Eo method employs horseradish peroxidase (HRP) to catalyze oxidative coupling using hydrogen peroxide (H_2_O_2_), to form di-tyrosine crosslinks.^36^ Di-tyrosine bond formation can also be achieved through a photochemical route, using Eosin Y as a photo-initiator that under light irradiation generates singlet oxygen forming the same di-tyrosine bonds.^37^ This method offers the opportunity to generate hydrogels with high mechanical strength and excellent biocompatibility but requires careful control of H_2_O_2_ concentration due to its potential cytotoxicity.^36,38^ In general, increasing the concentrations of H_2_O_2_ increases the crosslinking extent and enhances the mechanical properties of the hydrogel, while HRP controls the reaction kinetics.^39^ For the photochemical route, the concentration of Eosin Y primarily influences the efficiency of photo-crosslinking,^40^ although due to its capacity of penetrating cell membranes, excessive levels of Eosin Y may compromise cell viability. Both methods can be combined to control the overall level of crosslinking density, and hence the final mechanical properties. Therefore, careful optimization of these components is essential to achieve hydrogels with desirable physicochemical properties and biocompatibility.

More recently, a common way of generating functional dECM-containing constructs was introduced by the implementation of an SPS/Ru crosslinking reaction, allowing for the overall retention of stability, as well as functionality of the proteins residing within the developed constructs.^41–43^ This method provides precise crosslinking under light control with lower cytotoxicity;^44^ however, exposure to light and free radicals generated during the reaction may potentially damage encapsulated cells. The concentrations of SPS and Ru are critical in regulating the crosslinking dynamics and the resulting properties of THA hydrogels.^45,46^ Elevated SPS levels increase the number of sulfate radicals available not only for tyramine coupling, thereby controlling the overall crosslinking density and stiffness (storage modulus) of the THA hydrogel network, but in principle non-selectively affect embedded proteins. In contrast, higher concentrations of the Ru(ii) photocatalyst, together with light intensity, primarily accelerate the activation of persulfate and radical generation, controlling the gelation kinetics and promoting the formation of a more uniform hydrogel network, or inter-protein binding. Nevertheless, excessive concentrations of either component may lead to over-crosslinking, reduced porosity, and increased cytotoxicity.^47^ Therefore, the chosen SPS and Ru concentrations provide a controlled gelation process, yielding hydrogels with appropriate mechanical performance and biocompatibility, but posing non-selectivity in reactions with other embedded molecules, such as proteins.

Surface exposed tyrosine residues have indeed been shown to allow grafting and covalent interactions to proteins, such as those embedded in dECM,^48^ paving the way for evaluating the potential influence of tyramine-modified HA as binder matrix for the functional dECM. In this context, we systematically compared two crosslinking approaches—HRP/Eo with H_2_O_2_ and SPS/Ru—to fabricate THA-dECM hydrogels, aiming to evaluate their physicochemical properties, cytocompatibility, and suitability for cartilage regeneration. Particular attention was given to investigating the influence of the chosen crosslinking method on gelation kinetics, morphological features of the formed composite networks, mechanical performance, and chondrocyte phenotype. This work aims to fill a knowledge gap regarding the optimal crosslinking approach for THA-based hydrogels incorporating dECM for cartilage regeneration.

## 2. Methods

### 2.1. Synthesis of tyramine modified hyaluronic acid (THA)

The THA was synthesized using a previously described method.^15,49,50^ HA sodium salt was dissolved at 1 w/v% in ultrapure water. A one-step reaction was then performed by addition of 1.25 mmol 4-(4,6-dimethoxy-1,3,5-triazin-2-yl)-4-methylmorpholinium chloride (DMTMM) coupling agent, and subsequent dropwise introduction of 1.25 mmol tyramine hydrochloride to the solution. The reaction was carried out at 37 °C under continuous stirring for 24 h. The product was purified *via* precipitation with 96% ethanol after the addition of 10 v/v% saturated sodium chloride. ^1^H Nuclear magnetic resonance spectroscopy (NMR) was measured on Bruker Avance III (300 MHz) and used to verify the successful grafting of tyramine on the backbone with the appearance of the characteristic resonances at 7.17–7.19 ppm, 6.85–6.86 ppm, and 7.36–7.38 ppm. The degree of substitution was determined to be 6.2% based on absorbance readings at 275 nm on Infinite® 200 Pro plate reader (Tecan, Männedorf, Switzerland) using tyramine hydrochloride as the standard.

### 2.2. Preparation of dECM microparticles

For preparation of dECM, articular cartilage was collected from bovine stifle joints (aged 276–380 days) within 48 hours post-slaughter. The cartilage tissue was harvested by exposing the knee joint cavity and using a scalpel to collect full-thickness cartilage slices, ensuring that no calcified tissue was included.^15^ The harvested tissue fragments were washed with phosphate-buffered saline (PBS, Sigma Aldrich, USA) and immediately frozen at −20 °C. After five freeze/thaw cycles (−20 °C for 16 hours/room temperature for 8 hours) and pulverization in a liquid nitrogen mixer mill (MM 400, Retsch) at a frequency of 25 Hz for 3 minutes, the pulverized tissue was treated with DNase I (100 U mL^−1^, Sigma Aldrich, USA) and a protease inhibitor cocktail (0.2% v/v, Sigma Aldrich, USA) for 8 hours at 37 °C to remove cellular DNA.^51,52^ Subsequently, the decellularized tissue was lyophilized, followed by a second pulverization in the nitrogen mixer mill (MM 400, Retsch) at a frequency of 25 Hz for 15 minutes.

### 2.3. Preparation of decellularized extracellular matrix (dECM) containing THA hydrogels

To prepare the THA-dECM hydrogel, 3 w/v% THA was first dissolved in PBS and stirred at 4 °C for 24 hours to ensure complete dissolution. For HRP/Eo crosslinking, PBS containing 0.1 U mL^−1^ horseradish peroxidase (HRP; Sigma-Aldrich, USA) was prepared. In contrast, for SPS/Ru, THA was dissolved in plain PBS without HRP. After complete dissolution of THA, dECM particles were incorporated to avoid aggregation and ensure homogeneous dispersion. Based on previous experiments, 20% (w/w relative to THA) dECM particles were added to the THA solution, and the mixture was gently stirred at room temperature until uniform.^15^ The resulting THA-dECM precursor solutions were then subjected to crosslinking using either the HRP/Eo or SPS/Ru method.

#### 2.3.1 Horseradish peroxidase/Eosin (HRP/Eo) method

Enzymatic crosslinking was initiated by adding 0.15 mM H_2_O_2_ to the precursor solution in custom-made 3D molds (6 mm diameter and 2 mm height), followed by incubation at 37 °C for 30 minutes. Subsequently, additional photochemical crosslinking was carried out by exposing the solution to green light (518 nm) at room temperature for 30 minutes, using Eosin Y (0.2 mg mL^−1^; Sigma Aldrich, USA) as the photoinitiator. Hydrogels were exposed to light from a Eurolite LED IP FL-30, emitting at 518 ± 10 nm, with a working distance of 10 cm.^15^ The HRP/Ru system exhibits more stable and controllable network formation due to the synergistic effect of enzymatic and photochemical crosslinking (Fig. S1).

#### 2.3.2 Persulfate/ruthenium (SPS/Ru) method

The SPS/Ru photocrosslinking process was initiated by preparing the THA hydrogel solution at 3 w/v% with a final concentration of 0.1 mM ruthenium II trisbipyridyl chloride ([RuII(bpy)_3_]^2+^) (Ru) and 4 mM Sodium Persulfate. Ru powder was dissolved in PBS at a concentration of 37.4 mg mL^−1^, and Sodium Persulfate was prepared similarly at a concentration of 119 mg mL^−1^. The required volumes of each stock solution were sequentially incorporated into the pre-hydrogel formulation. Pipette mixing was performed thoroughly after each addition to ensure homogeneity, while avoiding direct interaction between ruthenium and sodium persulfate prior to their incorporation into the hydrogel solution. The mixture was then exposed to visible blue LED light (400–450 nm) at an intensity of 50 mW cm^−2^ for 3 minutes at room temperature to achieve photocrosslinking. Light irradiation was performed using an Eurolite LED IP FL-30.^45^

### 2.4. Swelling and degradation assays

For swelling and degradation studies, the hydrogel precursor solutions were cast and photocross-linked as described above (Methods 2.3) in molds with a diameter of 6 mm and a height of 2 mm. The freshly prepared gels were weighed using an electronic Semi-Micro Balance (Mettler Toledo) before being transferred to TPP tissue culture plates (Sigma-Aldrich), immersed in 2 mL of PBS, and incubated at 37 °C to allow swelling. At predefined time points (2, 4, 6, 24, 48, and 72 h), hydrogels were gently blotted with tissue paper to remove surface liquid and weighed to determine their wet weight (*W*_*t*_) at time *t*. *W*_0_ was noted as the initial weight after photocross-linking. For degradation assays, hydrogels were first allowed to reach swelling equilibrium in PBS at 37 °C for 24 h. The equilibrium weight was recorded as the swollen weight (*W*_swollen_). The samples were then transferred into fresh PBS containing 100 U mL^−1^ hyaluronidase (Sigma-Aldrich), 1 mg mL^−1^ collagenase II, or a mixture of both enzymes, and incubated at 37 °C. At predefined time points (2, 4, 6, 24, 48, and 72 h), the samples were retrieved, gently blotted to remove excess surface fluid, and weighed to determine the residual mass. The degradation ratio was calculated as:degradation ratio = (1 − *W*_*t*_/*W*_swollen_) × 100%

All experiments were conducted with *n* = 6 independent replicates per formulation.

### 2.5. Fluorescence imaging to visualize hydrogel microstructure and matrix distribution

To visualize the microstructure and dECM distribution within hydrogels, fluorescein isothiocyanate–dextran (FITC-dextran, 0.1 wt%, *M*_w_ ≈ 2 MDa) was added to the hydrogel solution prior to crosslinking. For imaging, hydrogels were prepared as described above (Methods 2.3.) and imaged using an upright confocal microscope (LSM 800, Carl Zeiss AG) equipped with a 488 nm laser and a 5× objective lens. Ten single-plane images and one Z-stack (60 µm depth, 20 µm step size) were acquired from randomly selected regions for each condition. In addition, fluorescence intensity thresholding was applied to quantify dark (pixel intensity < 40) and bright (pixel intensity ≥ 40) regions within the hydrogel structure. Between 8 and 10 images per group were analyzed for quantitative evaluation.

### 2.6. Rheological testing of hydrogels

The rheological properties of the hydrogels were characterized using an Anton Paar MCR302 rheometer equipped with a parallel plate configuration (PP-25 plate, 25 mm diameter) and a gap distance of 1 mm. Storage modulus (*G*′) and loss modulus (*G*″) were measured to evaluate the gelation process, mechanical stability, and viscoelastic behavior of the hydrogels. For gelation kinetics, time sweep tests were performed at 37 °C with a constant oscillating strain of 1% and a frequency of 1 Hz. Amplitude sweep tests were performed to determine the linear viscoelastic region (LVR), using a strain range of 0.01% to 1000%. Frequency sweep tests were performed in the range of 0.1 Hz to 100 Hz at 1% strain within the linear viscoelastic region. All measurements were performed on freshly prepared hydrogels, and the effects of dECM incorporation and different cross-linking methods on the rheological properties were evaluated by comparing hydrogels with and without dECM and prepared by both above-described methods. To prevent sample dehydration during measurements, a thin layer of silicone oil was applied around the periphery of the sample. The tests were repeated at least three times for each hydrogel formulation.

### 2.7. Fourier transform infrared spectroscopy (FTIR) analysis of hydrogels

FTIR spectroscopy was employed to analyze the chemical structure and crosslinking characteristics of the hydrogels. Samples were first lyophilized and then mechanically ground into fine powder for analysis. Spectra were acquired using a spectrometer equipped with an attenuated total reflectance (ATR) accessory (FT/IR-4X, JASCO), scanning in the range of 4000–400 cm^−1^. All samples were measured in a dry state, and the air background spectrum was subtracted prior to analysis. This analysis aimed to evaluate the involvement of dECM in the crosslinking process and to compare hydrogels formed *via* different gelation methods (*i.e.*, HRP/Eo and SPS/Ru), thereby assessing the effects of formulation and crosslinking strategy. Each spectrum was recorded at a resolution of 4 cm^−1^ and averaged over 32 scans. All measurements were performed in triplicate for each sample, and the data were normalized prior to analysis. The intensity ratio of two characteristic peaks (*I*_1630_/*I*_1030_) was calculated and presented in the Results section.

### 2.8. *In vitro* 3D culture of THA-dECM hydrogels encapsulated with chondrocytes

Cartilage tissue was collected from bovine stifle joints post-slaughter and washed with sterile PBS. The cartilage tissue was cut into small pieces and incubated in DMEM medium containing 0.1% pronase for 105–120 minutes at 37 °C. This was followed by digestion with 600 U mL^−1^ collagenase II overnight (12–16 hours) at 37 °C to isolate chondrocytes. After digestion, the cell suspension was filtered through a 70 µm cell strainer to remove undigested tissue fragments. The isolated chondrocytes were seeded into T300 flasks at a density of 5 × 10^6^ cells per T300 flask. The culture medium used was high-glucose DMEM supplemented with 10% fetal bovine serum (FBS) and 1% penicillin-streptomycin (Pen-Strep). When the cells reached approximately 80–90% confluency, they were detached with 0.25% trypsin–EDTA solution and collected. The cells were passaged at a density of 5 × 10^6^ per T300 flask twice to obtain passage 3 chondrocytes. Chondrocytes were resuspended directly in the hydrogel precursor solution immediately prior to crosslinking using the molds described above, at a final density of 5 × 10^6^ cells mL^−1^. This approach ensured homogeneous cell distribution without altering the hydrogel concentration. Chondrocytes from three independent bovine donors were used, and THA–dECM hydrogels were prepared using three different batches of dECM materials.^15^ After crosslinking, the samples (6 mm diameter and 2 mm height cylinder shape) were cultured in DMEM supplemented with 1% insulin–transferrin–selenium (ITS), 1% ascorbate-2-phosphate, and 1% non-essential amino acids (NEAA) for 1, 7, or 14 days.

### 2.9. Biochemical analysis of THA-dECM hydrogels

For biochemical analysis, THA-dECM hydrogels were first digested with 1 mL of hyaluronidase (1000 U mL^−1^) for 24–48 hours at 37 °C in a rotator. After digestion and centrifugation, the supernatant was transferred into fresh tubes and the remaining precipitate from hyaluronidase digestion was further digested with 1 mL of Proteinase K (1 mg mL^−1^) at 56 °C overnight. The digestion solution from hyaluronidase and proteinase K were combined for biochemical testing. DNA quantification was carried out using the PicoGreen assay (Thermo Fisher, USA) according to the manufacturer's instructions. GAG concentration was quantified using the 1,9-dimethylmethylene blue (DMMB, Sigma Aldrich) assay at a wavelength of 535 nm. A standard curve was generated using chondroitin sulfate (Sigma Aldrich, USA). Collagen concentration was measured with hydroxyproline assay after NaOH hydrolysis (Sigma Aldrich, USA) and neutralization with citric acid (Sigma Aldrich, USA).

### 2.10. Safranin O/fast green staining

The THA-dECM hydrogels were embedded in tissue freezing medium (Leica, Germany) and rapidly frozen. The frozen samples were sectioned into 10 µm slices using a cryostat (Leica, Germany) for histological staining. After sequential fixation with 70% methanol and 100% methanol, the slides were stained with 0.1% Safranin O in 0.1% acetic acid (Sigma Aldrich, USA) and 0.02% Fast Green (Sigma Aldrich, USA) to observe the distribution of proteoglycans (PG) and collagen. Weigert's hematoxylin (Merck, USA) was used for counterstaining to observe cell nuclei. Safranin O/Fast Green-stained sections were imaged under identical microscope settings. PG staining intensity was quantified using ImageJ software by measuring the integrated density of the red channel in defined regions of interest (ROIs). At least three sections per sample and three randomly selected fields per section were analyzed, and the mean value was used for statistical comparison.

### 2.11. Gene expression analysis

Total RNA from hydrogels was isolated using TRI Reagent (Molecular Research Center, MRC). Each hydrogel was lysed in 1 mL TRI Reagent and homogenized by a tissue lyser. After centrifugation, 0.1 mL 1-bromo-3-chloropropane (BCP) was added to the supernatant for phase separation. The aqueous phase was then collected for RNA extraction with Qiagen Rneasy MINI kit. For reverse transcription, 0.4 µg RNA was reverse transcribed using Superscript Vilo cDNA Synthesis Kit (Thermo Fisher). qPCR was performed with QuantStudio™ 7 Real-Time PCR System (Applied Biosystems). Relative gene expression levels were calculated using the 2^−ΔΔCT^ method. RPLP0 was used as the reference housekeeping gene for normalization. The primer and probe sequences of the tested genes are listed in Table 1.

**Table 1:** Oligonucleotide primers and probes used for RT-qPCR^a^

Gene	Primer & probe	Sequence/catalogue number
RPLP0	Primer and probe mix	Bt03218086_m1
COL2	Forward primer (5’→3′)	AAG AAA CAC ATC TGG TTT GGA GAA A
	Reverse primer (5’→3′)	TGG GAG CCA GGT TGT CAT C
	Probe (5′FAM/3′TAMRA)	CAA CGG TGG CTT CCA CTT CAG CTA TGG
ACAN	Forward primer (5′ → 3′)	CCA ACG AAA CCT ATG ACG TGT ACT
	Reverse primer (5′ → 3′)	GCA CTC GTT GGC TGC CTC
	Probe (5′FAM/3′TAMRA)	ATG TTG CAT AGA AGA CCT CGC CCT CCA T
COL1	Forward primer (5′ → 3′)	TGC AGT AAC TTC GTG CCT AGC A
	Reverse primer (5′ → 3′)	CGC GTG GTC CTC TAT CTC CA
	Probe (5′FAM/3′TAMRA)	CAT GCC AAT CCT TAC AAG AGG CAA CTG C
SOX9	Forward primer (5′ → 3′)	AAC GCC GAG CTC AGC AAG
	Reverse primer (5′ → 3′)	ACG AAC GGC CGC TTC TC
	Probe (5′FAM/3′TAMRA)	TTC AGC AGT CTC CAG AGC TTG CCC A

a ACAN – Aggrecan, RPLP0 – Ribosomal Protein Lateral Stalk Subunit P0, COL2 – Collagen Type II Alpha 1 Chain, COL1 – Collagen Type I Alpha 1 Chain, SOX9 – SRY-Box Transcription Factor 9. Primers and probes presented with sequences were self-designed, while the one with catalogue number was gene expression assay from Applied Biosystems.

### 2.12. Compression test of THA-dECM hydrogels

Unconfined uniaxial compression tests were performed on hydrogels using an electromechanical testing machine equipped with a 50 N load cell (LTM1, ZwickRoell, Germany). For each group, nine samples were equilibrated in DMEM for 30 min prior to testing. A displacement rate of 0.5 mm min^−1^ was then applied until failure. The slope within the linear region corresponding to 10–20% strain was calculated as the compressive modulus.

### 2.13. Metabolic labelling of newly synthesized GAGs

In separate experiments, hydrogels were cultured in DMEM supplemented with 1% ITS, 1% ascorbate-2-phosphate, 1% non-essential amino acids and 50 µM tetraacetylated *N*-Azidoacetyl-galactosamine Ac_4_GalNAz (CLK-1086, Jena Bioscience).^53^ Ac_4_GalNAz metabolically replaces its natural counterpart, *N*-acetylgalactosamine, and introduces an azide group that enables subsequent labeling with a photoactive moiety. Ac_4_GalNAz was replenished with each medium change (every second day) from day 1 to day 14. The control group was cultured without Ac4GalNAz. After 14 days, hydrogels were rinsed in fresh DMEM for 2 hours, briefly washed with PBS, embedded in Tissue-Tek OCT, rapidly frozen, and sectioned into 10 µm slices. The sections were air-dried for 30–60 minutes, washed with PBS-T (0.15% Tween-20 in PBS), and incubated at room temperature for 30 minutes with a click reaction mixture containing 1 µM Sulfo-Cy5-Alkyne, 1 µg mL^−1^ DAPI, 4 mg mL^−1^ sodium ascorbate, and 0.5 mg mL^−1^ copper sulfate. After incubation, samples were washed twice with PBS-T for 5 minutes each, then mounted with coverslips and imaged using a BX63 motorized fluorescence microscope. Fluorescence intensity was quantified using ImageJ software by measuring the mean gray value within a circular region of interest (ROI) centered on each cell nucleus, with a fixed diameter of 15 µm. The gray value corresponds to the intensity of the red fluorescence channel. Background fluorescence was determined by measuring the mean intensity in a cell-free area of the same image and subtracted from each ROI measurement. For each sample, data from three sections and five randomly selected fields of view per section were averaged to calculate the results.

### 2.14. Hemolysis assay

Hemolysis assays were performed to evaluate the blood compatibility of the hydrogel formulations before and after crosslinking. Fresh anticoagulated sheep whole blood was collected and diluted with sterile saline to obtain a uniform erythrocyte suspension. Samples were incubated in PBS at 37 °C for equilibration and then immersed in the erythrocyte suspension. Triton X-100 and PBS were used as positive and negative controls, respectively. After incubation at 37 °C for 30 min, the samples were centrifuged at 1500 rpm for 5 min, and the supernatant was collected. The absorbance of the released hemoglobin was measured using a microplate reader at 540 nm.

The hemolysis ratio was calculated according to the following equation: hemolysis ratio (%) = [(*A*_sample_ – *A*_negative_)/(*A*_positive_ – *A*_negative_)] × 100%where *A*_sample_, *A*_negative_, and *A*_positive_ represent the absorbance of the sample, negative control, and positive control, respectively. All experiments were performed in triplicate.

### 2.15. Chondrocyte scratch (wound healing) assay

The migratory capacity of cells in response to hydrogel-conditioned environments was evaluated using a scratch assay. Bovine chondrocytes (P2, 5× 10^5^ cells per well) were seeded in 6-well plates and cultured until reaching approximately 90% confluence. A sterile 200 µL pipette tip was used to create a linear scratch across the cell monolayer. After washing with PBS to remove detached chondrocytes, fresh medium containing hydrogel extract or control medium (high-glucose DMEM) was added, with 1% FBS supplemented to each group.

Images of the scratch area were captured at 0 h and 24 h using an inverted microscope under identical magnification. The wound area was quantified using ImageJ software, and the percentage of wound closure was calculated as:wound closure (%) = [(*A*_0_ − *A*_*t*_)/*A*_0_] × 100%where *A*_0_ is the initial scratch area at 0 h and *A*_*t*_ is the remaining scratch area at 24 h. All experiments were performed with at least three independent replicates.

### 2.16. Statistical analysis

Statistical analysis was conducted using GraphPad Prism 10.1.2. The Shapiro–Wilk test was performed to assess the normality of data in each group. For data that followed a normal distribution, a one-way analysis of variance (ANOVA) with Šídák's multiple comparisons was carried out, whereas the Kruskal–Wallis test was applied to data that were not normally distributed. A *p*-value of less than 0.05 was considered to indicate statistical significance.

## 3. Results

### 3.1. Evaluation of THA-dECM hydrogel properties as a function of formulation composition

**Fig. 1:**
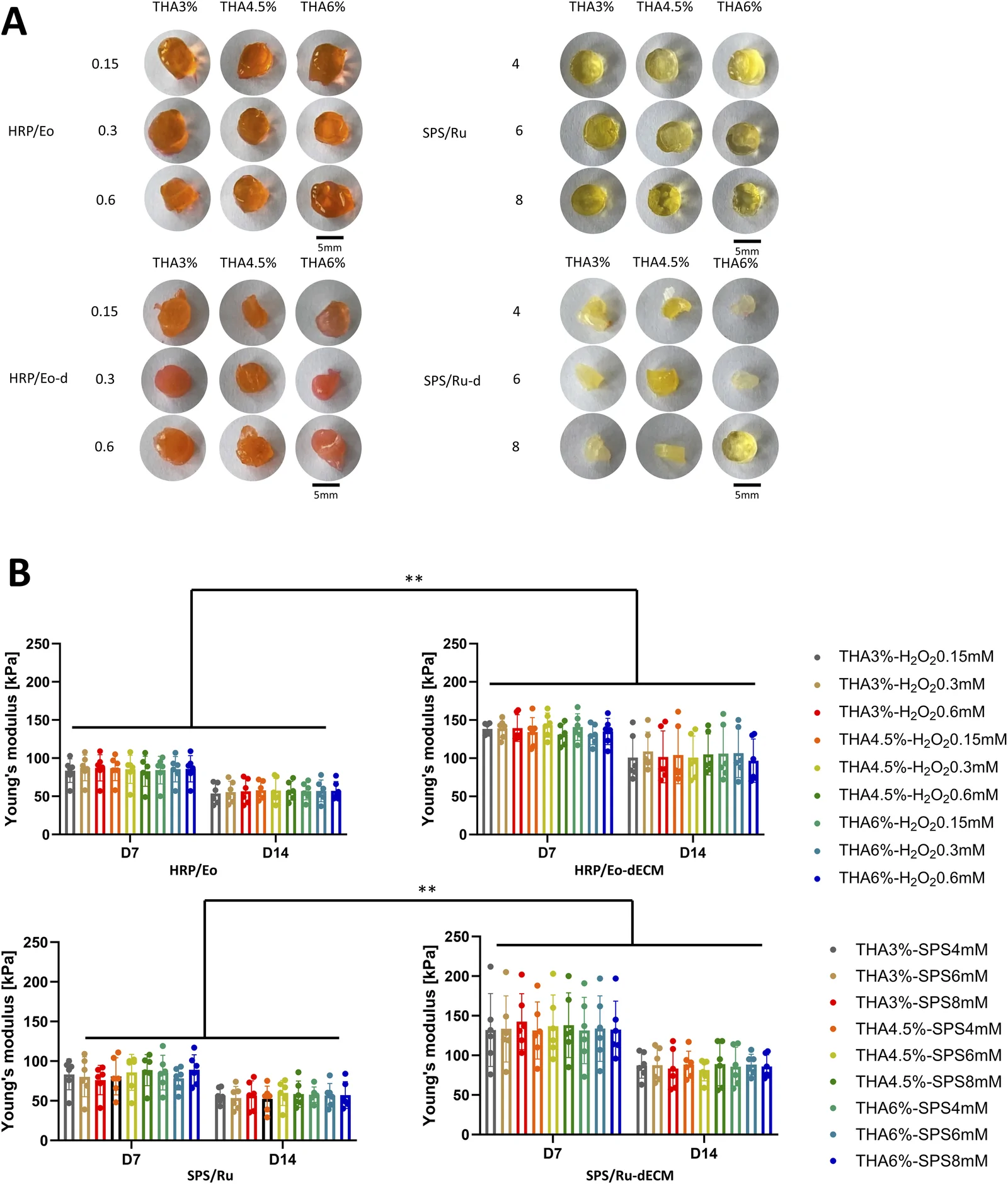
Assessment of THA hydrogels with varying concentrations of THA, H_2_O_2_ and SPS. (A) Macroscopic images of hydrogels on day 0. (B) Compressive modulus of hydrogels with different reagent concentrations on days 7 and 14. Hydrogels were incubated in DMEM medium without serum at 37 °C and 5% CO_2_; the medium was replaced every 2 days. Data presented as mean ± sd, *n* = 9. Statistical analysis was done using one-way analysis of variance (ANOVA) with Šídák's multiple comparisons; **p < 0.01.

In a pilot experiment, THA-dECM hydrogels were prepared at varying THA concentrations (3%, 4.5%, and 6%), and at variable crosslinkers/photo-initiator conditions (Fig. 1A). For horseradish peroxidase/Eosin (HRP/Eo)-crosslinked hydrogels, we tested different concentrations of H_2_O_2_ (1.5 mM, 3 mM, and 6 mM), at a fixed concentration of HRP (0.1 U mL^−1^), whereas for persulfate/ruthenium (SPS/Ru)-crosslinked hydrogels, SPS was tested at concentrations of 4 mM, 6 mM, and 8 mM at a fixed Ru concentration of 0.1 mM. The mechanical properties of obtained hydrogels were evaluated after 7 or 14 days of their incubation in DMEM (Fig. 1B).

The results showed that increasing the concentrations of H_2_O_2_ and SPS did not significantly increase the compressive modulus of the hydrogels at either of the measured days. However, the addition of dECM significantly increased the compressive modulus across all formulations (*p* < 0.01), irrespective of the cross-linking method used (Fig. 1B). We also observed a decrease in the overall compressive modulus for all formulations from day 7 to day 14, indicating that the THA-dECM hydrogels underwent swelling and physical degradation under aqueous incubation conditions. Considering that lower reagent concentrations have been shown to exert minimal effects on cell viability,^15,45^ and that there was no significant difference in compressive modulus between the two methods on days 7 and 14, we selected THA concentration of 3 w/v%, with 0.15 mM H_2_O_2_ for the HRP/Eo method and 4 mM SPS in SPS/Ru method, as the optimal crosslinker concentrations for the subsequent experiments described in this paper.

**Fig. 2:**
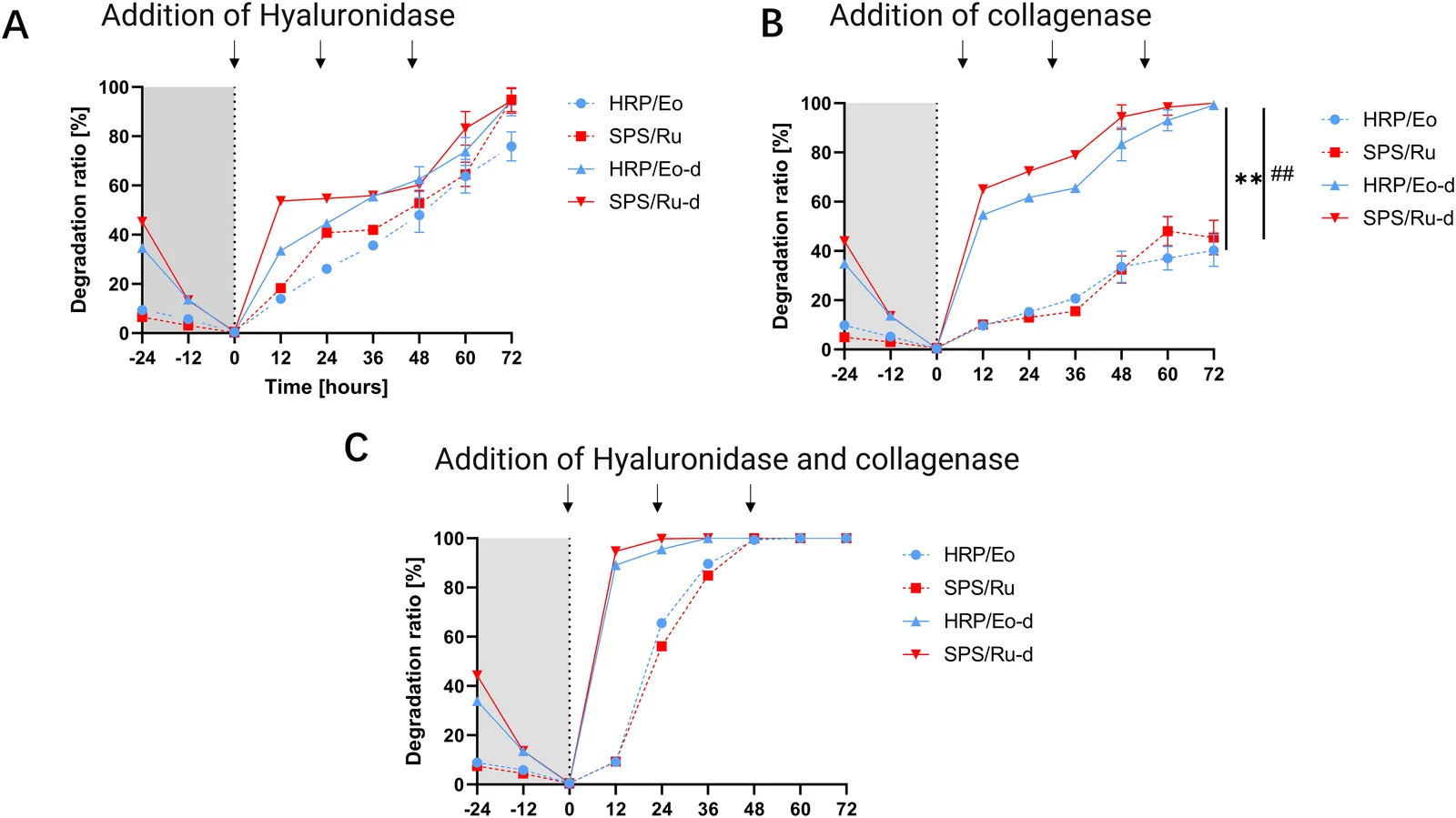
Degradation assays. The samples were transferred into fresh PBS containing 100 U mL^−1^ hyaluronidase (A), 1 mg mL^−1^ collagenase II (B), or a mixture of both enzymes (C), and incubated at 37 °C. Data are presented as mean ± sd (*n* = 6, the arrow represents the culture medium renewal). The shaded area indicates the 24 h equilibration period, during which the hydrogels reached equilibrium swelling prior to enzymatic degradation. Degradation was calculated relative to the sample weight at 0. Statistical analysis was performed using one-way analysis of variance (ANOVA) with Šídák's multiple comparisons test. Statistical significance was determined based on differences at the 72 h time point; **p < 0.01 HRP/Eo *vs.* HRP/Eo-d, ##*p* < 0.01 SPS/Ru *vs.* SPS/Ru-d.

Given the presence of both hyaluronic acid and dECM in our hydrogels, we subsequently performed degradation test using either hyaluronidase or collagenase, as well as their mixture, to evaluate the degradation effects of the enzymes from native tissue. Across all treatments, both HRP/Eo and SPS/Ru hydrogels exhibited similar behaviour (Fig. 2), however clear differences emerged in their sensitivity to enzymatic degradation and in the role of dECM. In Fig. 2A (hyaluronidase), all systems displayed similar linear degradation over 72 hours, with HRP/Eo hydrogels reaching 72.2 ± 7.4%, as compared to almost complete degradation of all other samples. Incorporation of dECM seemed to speed up the degradation response in both systems, with HRP/Eo-d and SPS/Ru-d reaching higher values at earlier time points (12 hours) than their respective control hydrogels, indicating that its presence within hyaluronan-rich components may increase susceptibility to hyaluronidase. In Fig. 2B (collagenase), clearly the hydrogels containing dECM degraded much faster, with 12 hours already leading to over 50% degradation, followed by the slowing-down response, most likely of the remaining hyaluronic acid-based networks. SPS/Ru hydrogels exhibited a more pronounced response than HRP/Eo up to 36 hours, suggesting greater sensitivity to collagen degradation. This may suggest that, in the SPS/Ru system, the embedded dECM particles play a more prominent role in maintaining network stability. On the other hand, the control hydrogels followed slower degradation rates reaching 42.3 ± 4.5%, at 72 hours with collagenase digestion, without any noticeable difference between the used cross-linking method. When a mixture of both enzymes was used, all formulations show a rapid and near-simultaneous increase to a plateau, with dECM containing samples almost matching in behaviour, and control hydrogels matching each other (Fig. 2C). In the combined treatment, dECM containing hydrogels were fully degraded at 24 hours, whereas non-dECM reached full degradation at 48 hours. The combined results from enzymatic degradation point out that SPS/Ru hydrogels are more responsive to enzymatic treatments than HRP/Eo, and the inclusion of dECM consistently amplifies both the rate and magnitude of degradation-related changes, particularly under collagenase-containing conditions. It hence suggests that SPS/Ru crosslinking method leads to different incorporation of dECM particles as compared to the HRP/Eo.

For the above-chosen formulations, rheological measurements were conducted to determine the storage modulus (*G*′) and loss modulus (*G*″) of the resulting hydrogels. For the HRP/Eo method, enzymatic gelation was conducted for the first 30 minutes, until a significant acceleration and increase of storage modulus was observed upon subsequent light activation. Importantly, we demonstrated the effects of each crosslinking method independently, *i.e.* HRP/H_2_O_2_ enzymatic alone *versus* Eosin light crosslinking, *versus* both HRP/H_2_O_2_ + Eosin. For dECM containing gels, all the methods effectively enable generation of hydrogels with similar storage modulus of ∼10 kPa (Fig. S1). However, the advantage of the combination of both is the easiness of handling. Following enzymatic crosslinking, the hydrogels typically become partly pre-crosslinked, hence enabling their easier handling, printing, or potentially injection to the relevant side, prior to the full photocrosslinking, which can maximize the crosslinking density and overall stability of the gel locally.

**Fig. 3:**
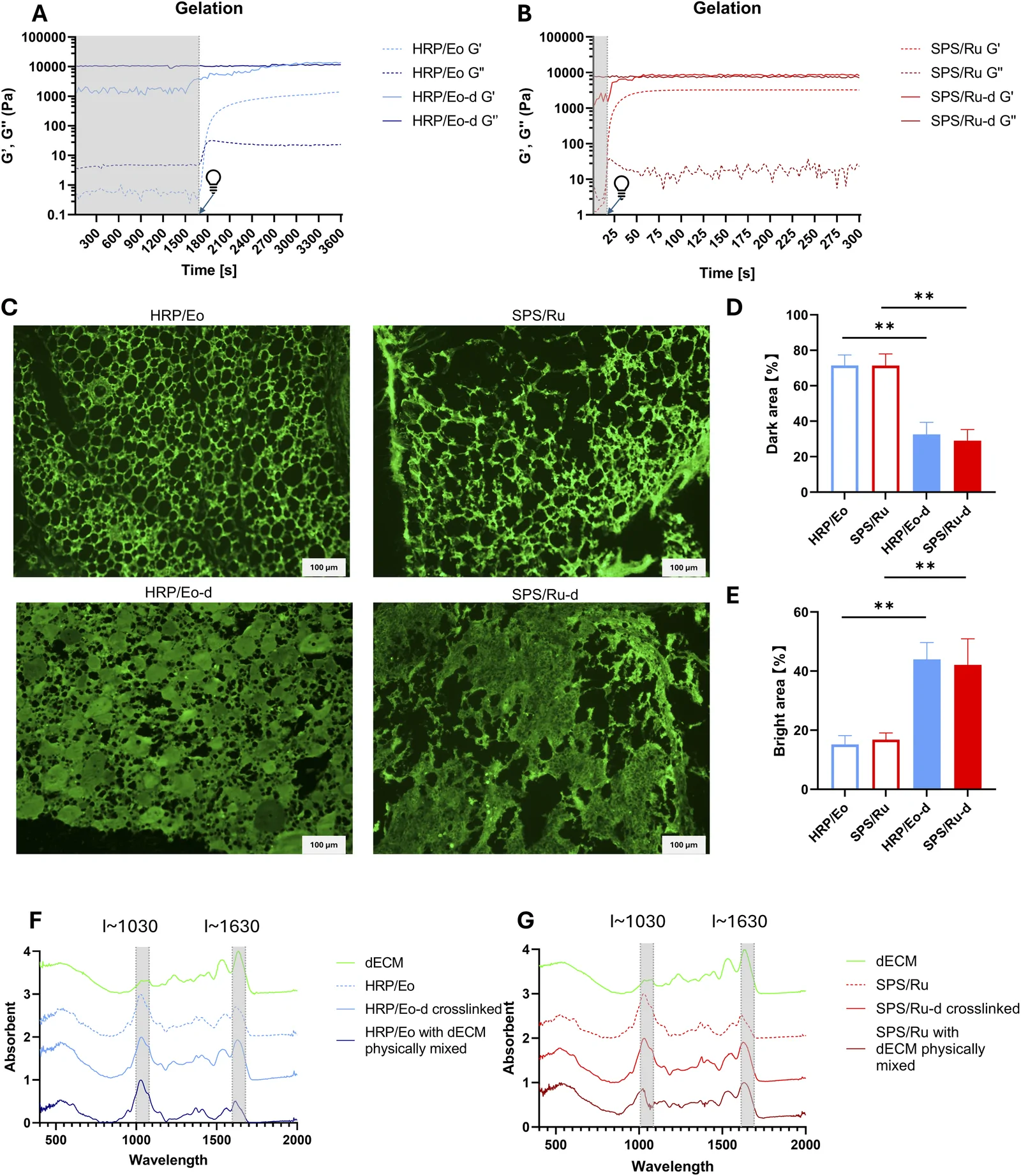
Rheological characterization, FTIR spectra and fluorescent tracer-based interstitial volume analysis of hydrogels prepared by HRP/Eo (0.15 mm H_2_O_2_) and SPS/Ru (4 mm SPS) crosslinking methods. (A and B) Gelation kinetics: the evolution of storage modulus (*G*′) and loss modulus (*G*″) over time for hydrogels formed by HRP/Eo (A) and SPS/Ru (B) systems. Interstitial matrix volume measurements in hydrogel. (C) 0.1 wt% fluorescein isothiocyanate–2 MDa dextran was added to each formulation prior to gelation and subsequent imaging. Dark (D) and bright (E) areas were calculated across *n* = 8–10 images for hydrogel microparticle composites. (F) FTIR spectra of hydrogels prepared using the HRP/Eo system, showing the characteristic peaks of dECM, HRP/Eo hydrogels, and dECM-incorporated HRP/Eo hydrogels: before gelation (HRP/Eo-d crosslinked) and after gelation (HRP/Eo with dECM physically mixed). (G) FTIR spectra of hydrogels prepared using the SPS/Ru system, showing the characteristic peaks of dECM, SPS/Ru hydrogels, and dECM-incorporated SPS/Ru hydrogels: before gelation (SPS/Ru-d crosslinked) and after gelation (SPS/Ru with dECM physically mixed). Data presented as mean, *n* = 3–5. Data are presented as mean ± sd (*n* = 8–10). Statistical analysis was done using one-way analysis of variance (ANOVA) with Šídák's multiple comparisons; *p* ** < 0.01.

The inclusion of dECM further increased both *G*′ and *G*″ (Fig. 3A), which can be attributed to the overall higher concentration of macromolecules (*e.g.*, collagen, glycosaminoglycans) that provide additional physical interactions and increase the viscoelasticity of the system. In contrast, the SPS/Ru method achieved gelation within 5 minutes, with *G*′ exceeding *G*″ within 20 seconds of light exposure. Without dECM, no significant differences in *G*′ and *G*″ were observed between the two methods; however, with dECM, the HRP/Eo group showed higher G' (11 200 ± 440 Pa) than the SPS/Ru group (7300 ± 350 Pa) (Fig. 3A and B). Amplitude sweep tests (Fig. S2A) identified a clear linear viscoelastic region (LVR, 0.01–60% strain) for both crosslinking methods, with *G*′ consistently exceeding *G*″, indicating solid-like behavior. G′ decreased beyond ∼91% strain, reflecting network failure. Gel–sol transitions (*G*′ = *G*″) occurred at ∼405% strain for HRP/Eo and ∼238% strain for SPS/Ru. Frequency sweeps (Fig. S2B) showed stable *G*′ > *G*″ over 0.1–39.8 Hz, confirming structural stability under physiologically relevant conditions.

In the subsequent hydrogel characterization step, confocal microscopy was performed to visualize the microstructure of the hydrogels. Both the THA network and dECM components were assessed using 0.1 wt% fluorescein isothiocyanate (FITC)-labeled dextran (2 MDa) as a high-molecular-weight fluorescent tracer to evaluate hydrogel morphology. Hence, the labelled (bright) regions depict both the THA matrix and dECM, whereas empty (dark) areas comprise of porous hydrated space. The incorporation of dECM can be clearly confirmed, specifically overlapping with the THA network (Fig. 3C), which is confirmed by lower dark and higher bright areas (Fig. 3D and E). Importantly, HRP/Eo-d clearly shows retention of much larger network-like feature surrounding particles, whereas SPS/Ru-d shows much more compact regions without so much surrounding THA matrix, indicating the morphological differences due to the differences in the used cross-linking mothods.

The scope of the FTIR analysis focused on evaluating the extent to which dECM can participate in these crosslinking processes and in di-tyrosine crosslinking in our formulations. First, we measured the FTIR of the control dECM, and control THA hydrogels prepared without any dECM. Next, the FTIR spectra were measured for two distinct cases: (i) where dECM was physically incorporated after the gel was crosslinked (named physically mixed in Fig. 3F and G); and (ii), where dECM was added prior to gelation, demonstrating our standard approach used throughout the article, which also represents the preparation method for biological studies presented in later sections. Given that dECM contains proteins, we hypothesize that FTIR spectra are dominated by the amide I peak centered around 1630 cm^−1^, which is present in the dECM,^54^ and to a lesser extend in hyaluronic acid-based polymers.^55^ In contrast, polysaccharides are typically characterized by prevalent intensity of the C–O stretching vibrations centered around 1031 cm^−1^.^55^ We hence focused on evaluating the outcome of participation of dECM based on the ratio *I*_∼1630_/*I*_∼1030_, shown for all above-described formulations in Table 2.

**Table 2:** Calculated *I*_∼1630_/*I*_∼1030_ values from FTIR spectroscopy. Colour shading is added to enable easier comparison between the two used crosslinking methods

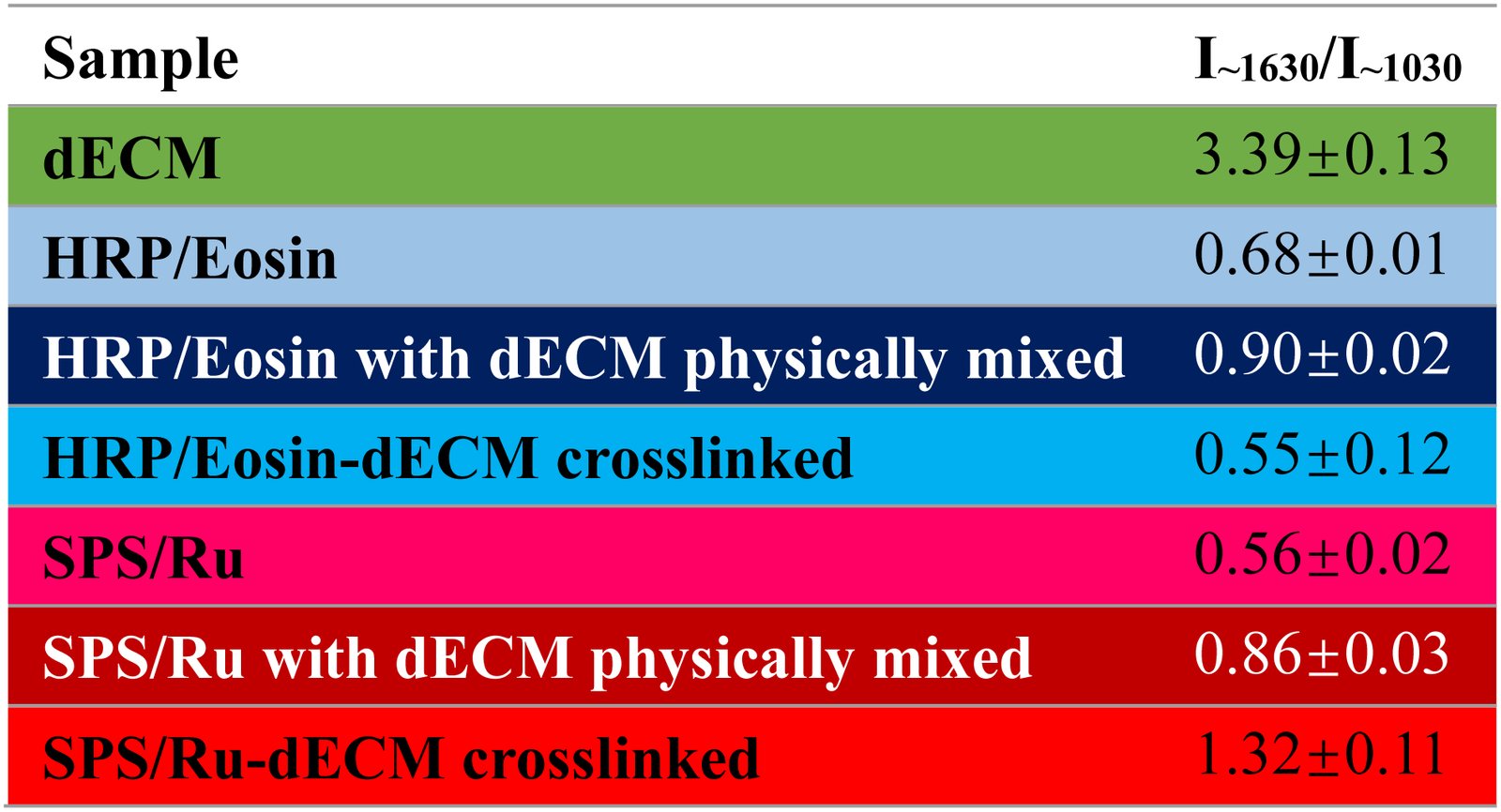

For dECM, the ratio, as expected, was highest, 3.39 ± 0.13, indicative of dominance of the region from the remaining proteins after the decellularization process. Both control hydrogels showed lower ratios dominated by carbohydrate vibrations, with slight method-dependent differences (0.68 ± 0.01 HRP/Eo *vs.* 0.56 ± 0.02 SPS/Ru). The SPS/Ru system exhibits stronger C–O stretching, whereas HRP/Eo adds minor amide contributions from its enzymatic pre-crosslinking step, accounting for the observed variation. When dECM was physically mixed into the hydrogels after crosslinking, both methods produced a similar band ratio of ∼0.9. This consistency indicates that dECM was incorporated only physically, with no active formation of di-tyrosine bonds between the dECM and the hydrogel components.

When dECM was present during crosslinking, the two methods produced distinctly different ratios, indicating that dECM participated differently in each reaction. In the HRP/Eo system, the reduced ratio (0.55 ± 0.12) may putatively suggest higher integration of dECM into the carbohydrate-rich network, whereas in SPS/Ru the increased ratio (1.32 ± 0.11) implies stronger exposure of protein-rich features *via* dominance of the amide band. These contrasting outcomes suggest that each method drives different degrees or modes of dECM involvement, leading to different protein exposure profiles in the final constructs, which coincide with the noted differences in degradation behaviour and observed morphological features in confocal microscopy.

**Fig. 4:**
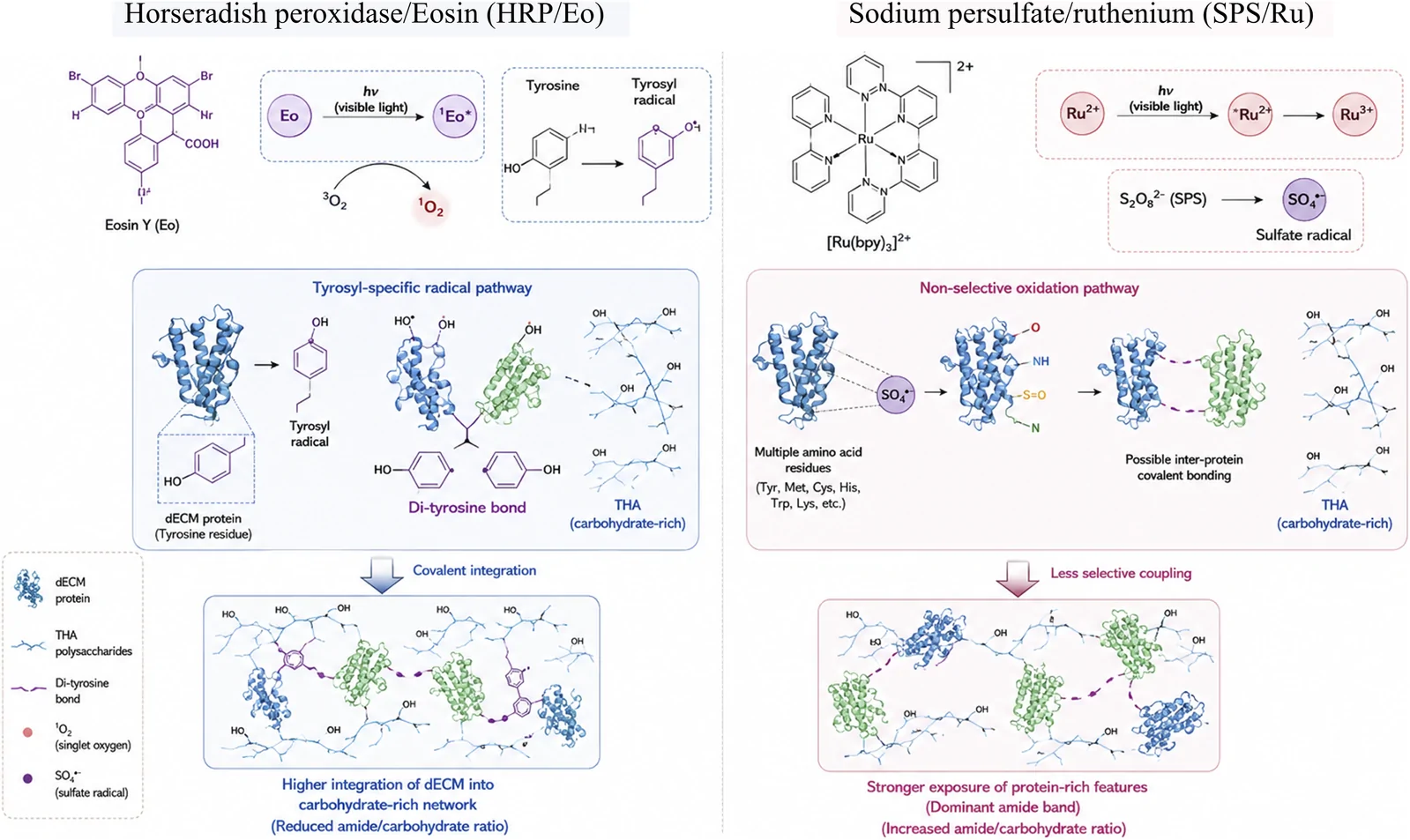
Proposed mechanisms of dECM participation during photochemical crosslinking using HRP/Eo and SPS/Ru systems.

These observations can be partly explained by the distinct radical species generated in each photochemical system. The Eosin Y photochemistry produces singlet oxygen (^1^O_2_) and tyrosyl-specific radicals that efficiently drive specific di-tyrosine bond formation, promoting covalent integration of dECM proteins into the THA network (Fig. 4). In contrast, the SPS/Ru system generates broad, highly reactive sulfate radicals (SO_4_˙–) that oxidize proteins in a less selective manner,^56^ potentially leading to reduced tyrosine-based coupling and possible inter-protein covalent bonding, and hence overall higher preservation or exposure of protein-rich features (Fig. 4). Consequently, the two crosslinking strategies engage dECM differently during gel formation, resulting in divergent molecular signatures in the final hydrogels (Fig. 4), which we relate to in the later sections of this manuscript.

### 3.2. Histological staining and DNA, GAG, collagen content

**Fig. 5:**
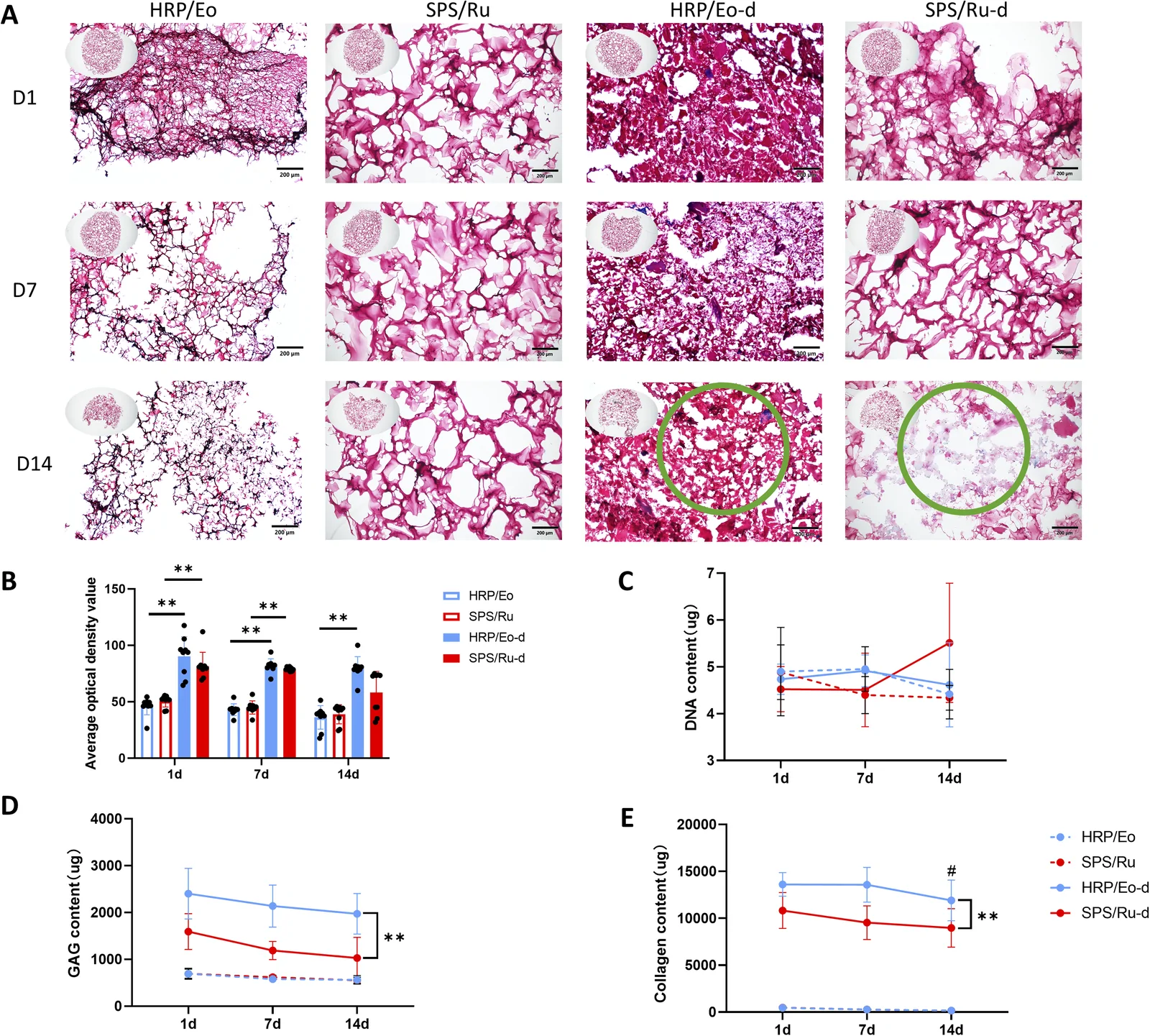
Characterization of hydrogels seeded with chondrocytes after 3D culture *in vitro*. (A) Safranin O/fast green staining on sections of hydrogels crosslinked using different methods. The green circles indicate the pore structures of hydrogels formed using the two different gelation methods. (B) Semi-quantification of optical density value. Scale bar = 200 µm. Content of DNA (C), gag (D) and collagen (E) in hydrogel during culture. Collagen content was calculated from hydroxyproline measurements using a conversion factor of 7.46, assuming hydroxyproline accounts for 13.4% of total collagen. Statistical analysis was done using one-way analysis of variance (ANOVA) with Šídák's multiple comparisons. Data presented as mean ± sd, *n* = 9, **p < 0.01.

Results from Safranin O/Fast Green staining (Fig. 5A) showed that, at the same time point, PG staining intensity was significantly higher in chondrocyte-laden, dECM-containing hydrogels than in chondrocyte-laden hydrogels without dECM. No significant differences in PG staining intensity were observed across different time points within the same group, indicating that the enhanced PG accumulation was primarily attributable to the incorporation of dECM rather than to temporal effects. After 14 days of culture, all groups maintained the integrity of the hydrogel structure. Notably, hydrogels prepared *via* the SPS/Ru method exhibited a more porous structure, whereas those fabricated using the HRP/Eo method showed a more granular morphology (Fig. 5A, green circle). On days 1 and 7, the dECM-containing groups in both methods showed significantly higher average optical density compared to their respective non-dECM controls. On day 14, no statistically significant difference was observed between the dECM and non-dECM groups in the SPS/Ru method, whereas a significant difference remained in the HRP/Eo method (Fig. 5A and B). DNA content indicated that the cell numbers in the hydrogels of all groups remained similar over the 14 day culture period (Fig. 5C). Furthermore, the GAG and collagen content in the hydrogels of all groups gradually decreased over time, reflecting the slow degradation of the hydrogels during the culture period. However, compared to other groups, the HRP/Eo-dECM group consistently maintained the highest levels of GAG and collagen (Fig. 5D and E). On day 14, HRP/Eo-dECM hydrogels retained 81.2% of their initial GAG content and 87.4% of their initial collagen content, indicating a relatively low degradation rate over the culture period. To further evaluate this, we measured the amount of GAG and collagen released into the conditioned medium during culture. The HRP/Eo-dECM group exhibited a slower release of GAG and collagen (Fig. S3), indicating more effective incorporation of dECM particles into the HRP/Eo-crosslinked network. Although no significant differences were observed between the two gelation methods in terms of cell migration performance, clearly higher wound closure was observed for samples that contained dECM (Fig. S4A and B). Hemolysis assay demonstrated that all hydrogels (both in solution and hydrogel form) were characterized by <5% hemolysis, indicating that the hydrogel formulations are considered non-hemolytic and hence safe for potential future *in vivo* testing (Fig. S4C).

### 3.3. Cartilage-related gene expression

**Fig. 6:**
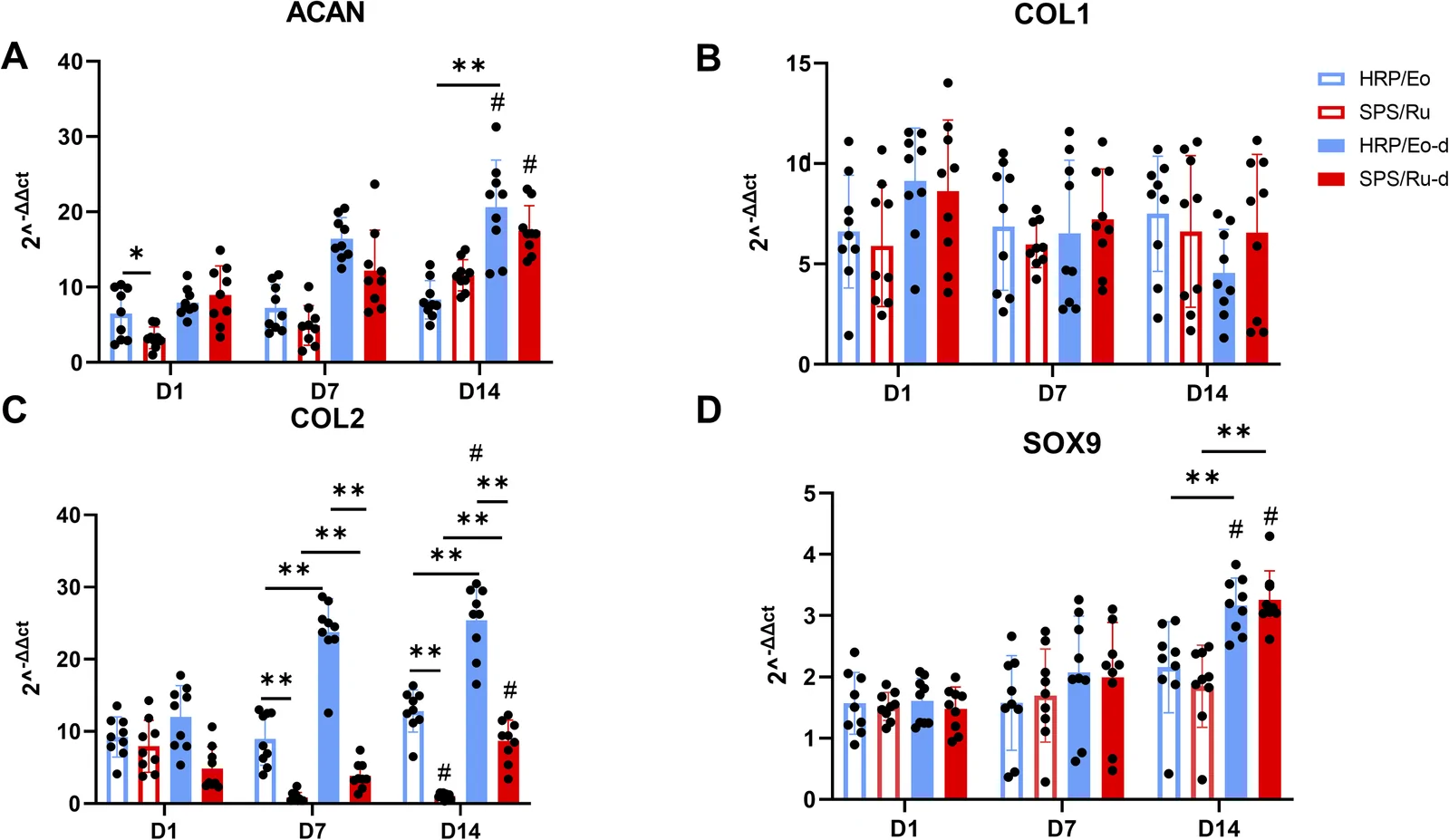
Fold change gene expression of (A) ACAN, (B) COL1, (C) COL2, and (D) SOX9, measured by RT-qPCR at day 1, 7 and 14 of culture on hydrogels from both methods, either containing dECM or without. Gene expression levels were normalized to P3 chondrocytes prior to seeding. Data presented as mean ± sd, *n* = 9, statistical analysis was done using Kruskal–Wallis test, *p < 0.05, **p < 0.01, #*p* < 0.05 *vs.* same group on day 1.

RT-qPCR was used to analyze the gene expression levels of chondrocytes in the four hydrogel groups at day 1, 7, and 14, with data normalized to the gene expression levels of P3 chondrocytes before seeding. The results showed that the expression of ACAN increased with the culture time, and dECM promoted the expression of ACAN in HRP/Eo hydrogels. However, no significant difference in ACAN expression was observed between the two methods on day 14 (Fig. 6A). The expression of COL1 stayed comparable with day 0 over time (Fig. 6B). Interestingly, the SPS/Ru-dECM group had the lowest COL2 expression level among the 4 groups on day 1, and during the 14 day culture period, COL2 expression gradually decreased in the SPS/Ru group. In contrast, in the HRP/Eo method, COL2 showed significant up-regulation by dECM incorporation and time-dependent increases. On day 14, the expression of COL2 in the HRP/Eo-dECM group was significantly higher than in the SPS/Ru-dECM group (Fig. 6C). For SOX9, there was no significant difference among the four groups on day 1, but the expression level of the dECM group increased in a time-dependent manner thereafter. On day 14, the expression level of SOX9 in the HRP/Eo-dECM group and the SPS/Ru-dECM group was significantly increased compared with the no-dECM group of the same method, but there was no significant difference in the expression level of SOX9 between the HRP/Eo-dECM group and the SPS/Ru-dECM group (Fig. 6D). Overall, the results suggest that the HRP/Eo method more effectively supports chondrocyte redifferentiation in the 3D culture model, as indicated by higher COL2 expression compared to the SPS/Ru method.

### 3.4. Mechanical properties

**Fig. 7:**
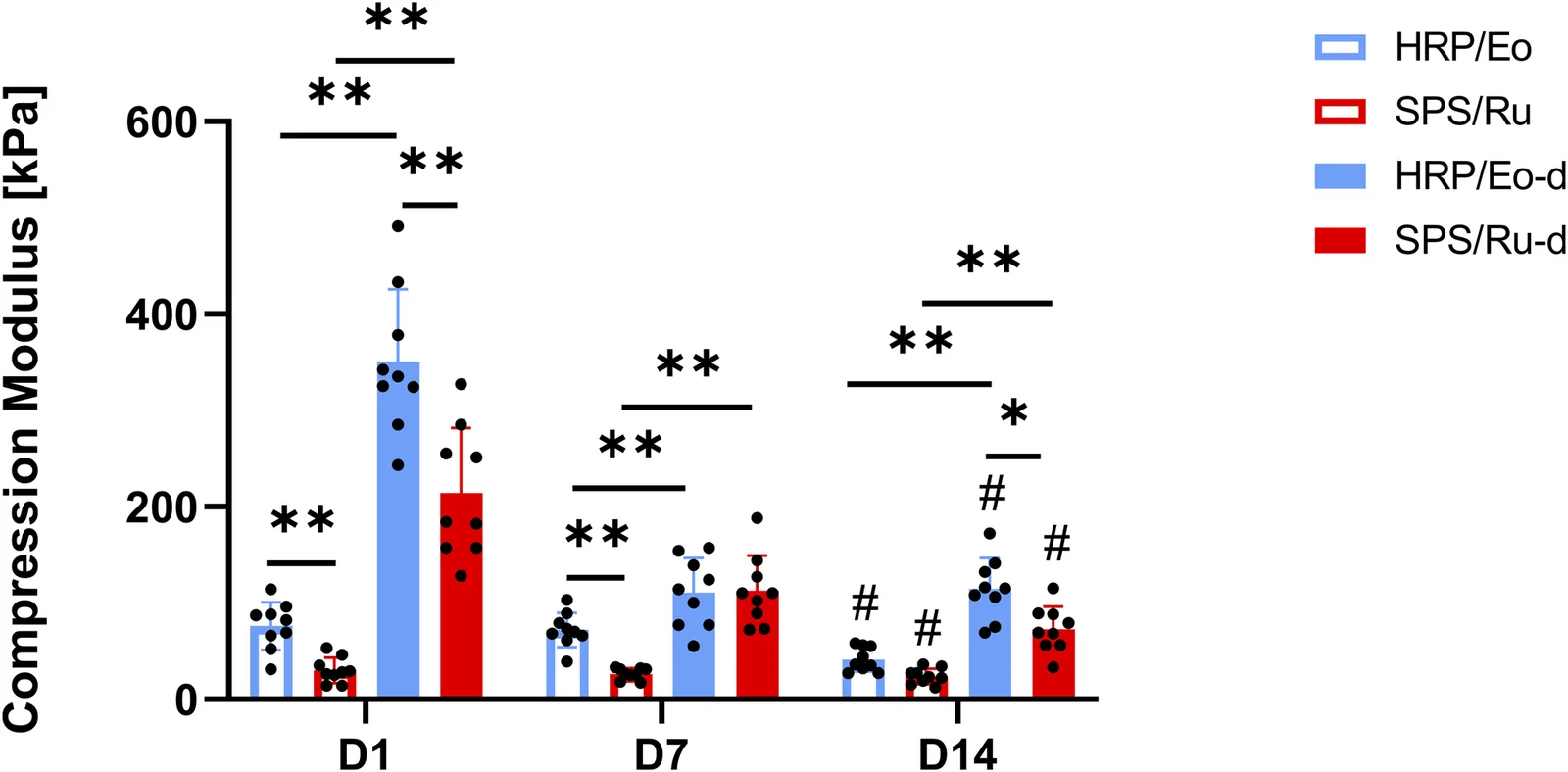
Characterization of hydrogel mechanical properties by compression test. Compressive modulus of hydrogels containing cells, with or without dECM cultured up to 14 days. Data presented as mean ± sd, *n* = 9, statistical analysis was done using one-way analysis of variance (ANOVA) with Šídák's multiple comparisons, *p < 0.05, **p < 0.01, #*p* < 0.05 *vs.* same group on D1.

The compressive modulus is a key mechanical parameter reflecting material stiffness. The results showed that cell-laden hydrogels exhibited the highest compressive modulus at the early stage of culture, whereas the compressive modulus of all four experimental groups decreased significantly after 14 days of culture (Fig. 7). Notably, the addition of dECM significantly increased the Young's modulus of hydrogels prepared by both methods. The HRP/Eo-dECM group reached 360 ± 80 kPa on day 1, while the SPS/Ru-dECM group reached 200 ± 40 kPa. The higher compressive modulus observed in HRP/Eo-dECM hydrogels after 14 days may be attributed to the more stable covalent network formed by the HRP/Eo crosslinking method, which resists hydrolytic and enzymatic degradation. The SPS/Ru-dECM hydrogels, by contrast, may be more susceptible to both hydrolytic cleavage and cell-mediated degradation, leading to a faster reduction in stiffness.

### 3.5. Newly synthesized GAG detection

**Fig. 8:**
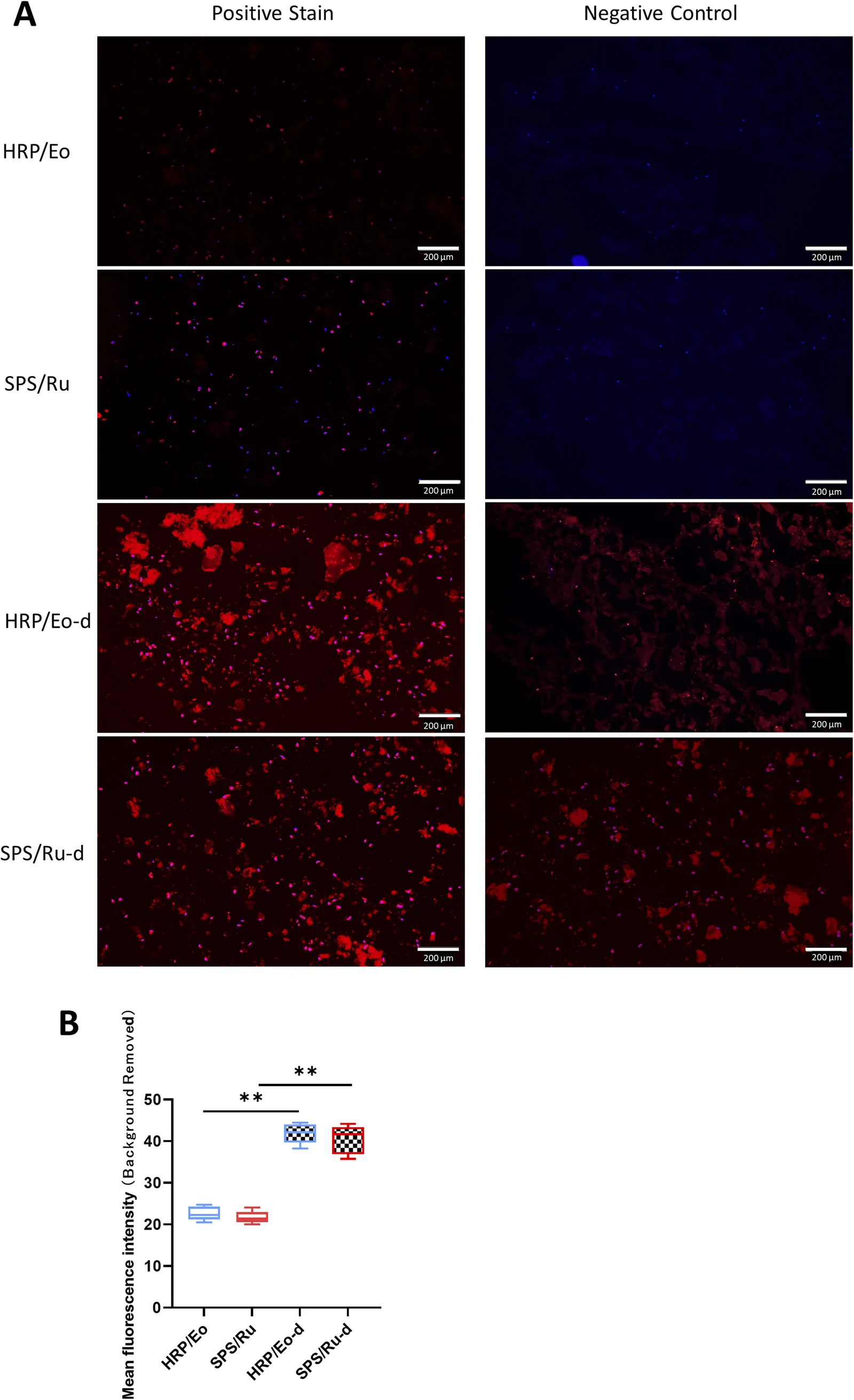
The spatial distribution and relative amount of newly synthesized GAGs in the HRP/Eo and SPS/Ru hydrogels with or without dECM cultured after 14 days. (A) Representative images of newly synthesized GAGs indicated by red staining, and the cell nuclei indicated by blue staining. Samples cultured without azide modified monosaccharides served as negative controls. (B) Staining intensity quantification of newly synthesized GAGs around the cells region. *n* = 9, statistical analysis was done using one-way analysis of variance (ANOVA) with Šídák's multiple comparisons, **p < 0.01.

To further quantify newly synthesized GAGs in the hydrogels, we used a metabolic labelling technique incorporating azide-modified sugars covalently labeled with a fluorescent probe *via* click chemistry for visualization and semi-quantification. Considering the strong fluorescent background of THA and dECM, we used samples without azide-modified monosaccharides as a negative control for background subtraction. DAPI was used to locate the cells, and the red fluorescence intensity around the cells was quantified and compared (Fig. 8A). The results showed that after 14 days of culture, dECM promoted the synthesis of new GAG by chondrocytes in the hydrogels prepared by both methods, although no significant difference was found between the two methods (Fig. 8B).

## 4. Discussion

Owing to their biomimetic composition and inherent bioactivity, dECM–based hydrogels have shown great promise in cartilage regeneration.^57–60^ However, insufficient mechanical stability during long-term culture remains a major obstacle to their clinical translation. Although increasing the hydrogel concentration or the concentration of crosslinking reagents can enhance mechanical properties, such approaches often compromise cellular bioactivity.^23,61–64^ To address these limitations, the present study systematically compared two crosslinking strategies—horseradish peroxidase/Eosin (HRP/Eo) and persulfate/ruthenium (SPS/Ru)—to optimize the gelation of THA–dECM hydrogels. Fig. 1 shows that increasing the catalyst concentration in either system failed to significantly enhance the Young's modulus of the hydrogels. This may be attributed to the fact that the polymer network had already approached its maximum crosslinking density, such that further increases in catalyst concentration could not generate a substantial number of additional crosslinking sites and therefore had only a limited effect on the overall stiffness. In contrast, as expected, irrespective of the crosslinking method employed, the incorporation of dECM consistently enhanced the compressive modulus of the hydrogels in our experimental system. Experimental results from chondrocyte-seeded hydrogels demonstrated that, under the tested conditions (3 w/v% THA and the selected SPS/Ru and HRP/Eo concentrations), HRP/Eo-dECM hydrogels exhibited higher compressive modulus and storage modulus (*G*′) during the early stage of culture. It should be noted that these mechanical values are specific to the chosen formulation and experimental parameters, and do not necessarily reflect the inherent mechanical superiority of one crosslinking method over the other. This difference may be associated with the dual enzymatic and photo-induced crosslinking mechanism of HRP/Eo, which could promote a denser or more interconnected network structure (Fig. S1). However, increased initial stiffness does not necessarily indicate superior long-term performance, as excessive crosslinking may also affect matrix diffusion, degradation behavior, or cell–matrix interactions over prolonged culture periods. Gene expression analysis further demonstrated that the incorporation of dECM substantially upregulated key cartilage-related genes (ACAN, COL2, and SOX9) under both crosslinking conditions, likely due to the retention of cartilage-specific biochemical cues within the matrix. Notably, COL2 expression was significantly higher in the HRP/Eo-dECM group than in SPS/Ru-dECM, which may indicate improved preservation of a chondrogenic microenvironment under the selected conditions. Nevertheless, these observations are based on specific hydrogel composition and crosslinking condition and therefore should not be generalized to all HRP/Eo or SPS/Ru formulations. Fluorescent imaging showed differences in morphology between HRP/Eo and SPS/Ru formulations containing dECM, where homogenously crosslinked network was obtained for HRP/Eo and aggregated dECM patches with THA were observed in the SPS/Ru case. This view was corroborated on molecular level by FTIR analysis, which revealed significant differences in gelation mechanisms. Compared with SPS/Ru–dECM hydrogels, HRP/Eo–dECM hydrogels exhibited a significantly lower *I*_1630_/*I*_1030_ ratio, matched with a reduced relative presentation of protein-associated dECM signals within the composite network. This observation suggests that dECM components are more uniformly embedded within the HRP/Eo-crosslinked hydrogel network. It may also reflect partial masking or altered presentation of amide-associated dECM signals resulting from the crosslinking process. These findings suggest that dECM interacts differently with the networks formed by each method: HRP/Eo crosslinking may promote homogenous integration between dECM and polysaccharide components, whereas SPS/Ru leads to self-association and SPS radical driven protein–protein binding, which generates topologically constrained networks. The homogenous integration in HRP/Eo hydrogel may facilitate slower release of bioactive matrix components, potentially contributing to improved biological performance. This was supported by measurements of GAG and collagen release into conditioned medium, which were lower in the HRP/Eo group, as well as the higher GAG and collagen retention within the HRP/Eo hydrogels. HRP/Eo hydrogels combine enzymatic and photo-induced crosslinking, which may offer additional flexibility in tuning gelation kinetics. This dual mechanism could potentially facilitate certain processing steps, such as embedding cells in pre-crosslinked and easier to handle biomaterial, before the final photocross-linking that may enable to reach high crosslinking density at any stage afterwards.

Despite these advantages, certain limitations remain. While HRP/Eo hydrogels demonstrated better mechanical properties under tested conditions and at tested concentrations at early time points, a marked decrease in compressive modulus was observed after 14 days of culture, indicating potential degradation issue for the long-term incubation.^65–67^ Further studies are needed to evaluate long-term mechanical stability and matrix retention for both HRP/Eo and SPS/Ru hydrogels.

## 5. Conclusions

In summary, this study provides a systematic comparison of two crosslinking strategies for fabricating THA-dECM hydrogels. Among the tested conditions, the HRP/Eo method consistently outperformed the SPS/Ru system. The dECM particles were homogenously incorporated *via* HRP/Eo-crosslinking and showed lower *I*_∼1630_/*I*_∼1030_ ratio from FTIR analysis, higher GAG and collagen retention, as well as slower GAG and collagen release, indicative of more effective incorporation in the molecular network. In agreement with this, confocal imaging revealed that HRP/Eo-dECM hydrogels retain a more extended network-like structure surrounding the dECM particles, whereas SPS/Ru-dECM hydrogels exhibited more compact regions with less surrounding THA matrix, confirming differences in morphology arising from the chosen crosslinking mechanism. Consistently, enzymatic degradation studies demonstrated that SPS/Ru hydrogels are more responsive to enzymatic treatments, while HRP/Eo hydrogels degrade more slowly. Moreover, the incorporation of dECM amplified the rate and magnitude of degradation, particularly under collagenase-containing conditions, reflecting the known differences in dECM integration within the network. As a result, HRP/Eo-dECM hydrogels exhibited markedly higher mechanical stiffness and chondrogenic gene expression level, highlighting their superior biomechanical and biological property. Collectively, our findings identify the HRP/Eo crosslinking strategy—specifically when combined with bioactive dECM particles—as a promising approach to enhance the biochemical activity and regenerative potential of dECM-based hydrogels for cartilage repair.

## Conflicts of interest

There are no conflicts of interest to declare.

## Supplementary Material

RA-OLF-D6RA01580H-s001

## Data Availability

All the data from this work will be available upon request addressed to the corresponding author. Supplementary information (SI): additional figures. See DOI: https://doi.org/10.1039/d6ra01580h.
