# Supplementary material for: Crosslinking strategies govern the morphology and biological performance of decellularized extracellular matrix particle–tyramine-modified hyaluronic acid hydrogels
Source: RSC Adv. 2026 Aug 24;16(44):54649–66. doi: 10.1039/d6ra01580h (PMC13501466; doi:10.1039/d6ra01580h)
Supplement: Supplementary file 1 [file RA-016-D6RA01580H-s001.pdf]

Supplementary Information (SI) for RSC Advances.
This journal is © The Royal Society of Chemistry 2026

Supplementary Information

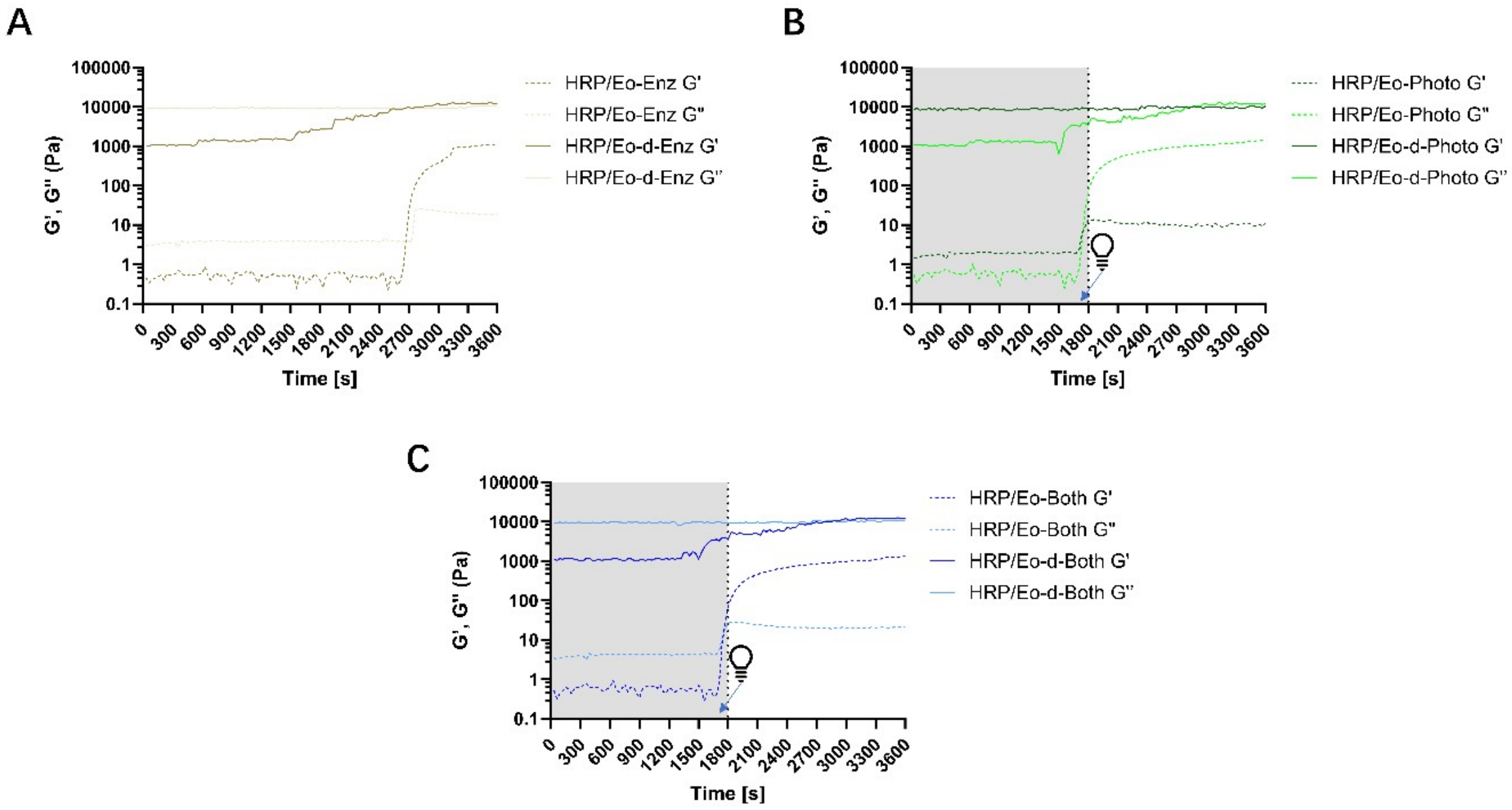

Fig. S1. Rheological characterization of hydrogels prepared via HRP/Eo crosslinking. Gelation kinetics show the evolution of storage modulus (G′) and loss modulus (G″) over time for hydrogels formed by enzymatic crosslinking (A), photochemical crosslinking (B), and the combined enzymatic/photochemical system (C). Data presented as mean, n = 3-5.

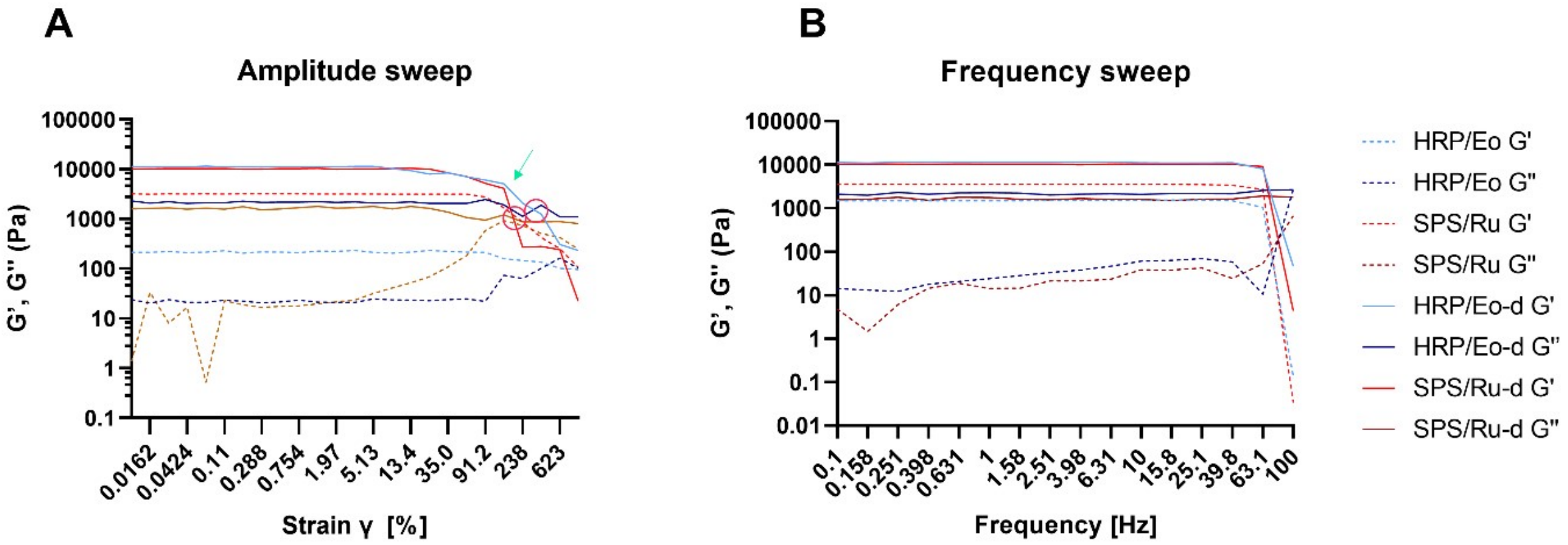

Fig. S2. Amplitude and Frequency sweep of hydrogels. (A) Amplitude sweep: Dependence of G' and G'' on strain (γ) for hydrogels, showing the linear viscoelastic region (LVR) and the strain at which the network structure starts to break（Green arrow）. (B) Frequency sweep: Stability of the hydrogels over a range of frequencies, demonstrating consistent viscoelastic behavior across different crosslinking systems. Data presented as mean, n = 3-5.

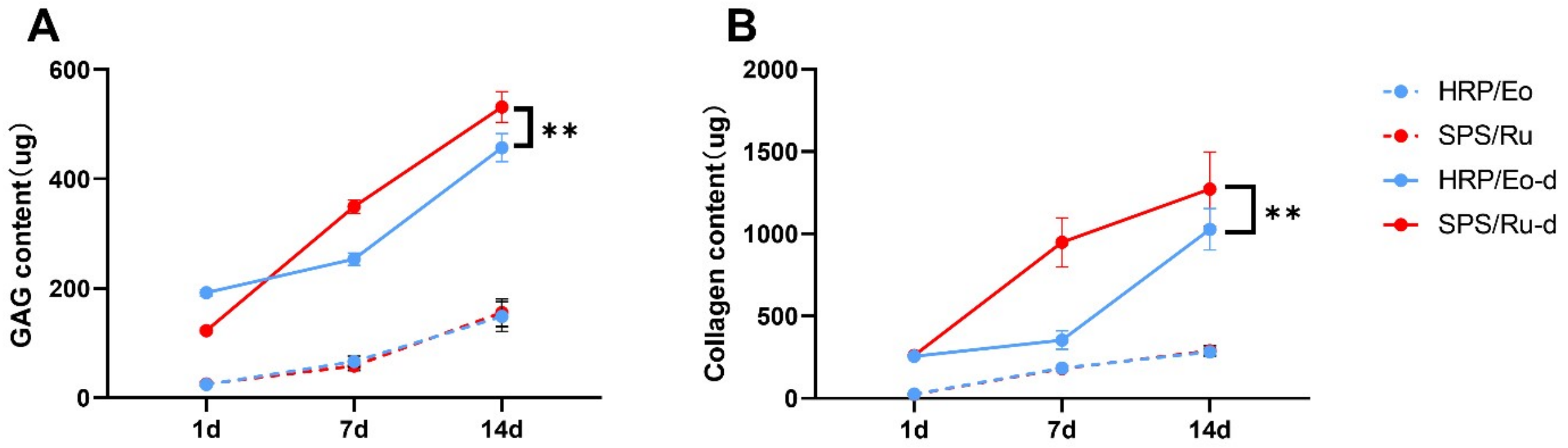

Fig. S3. The release of GAG and collagen. Data presented as mean + SD, n = 9, Statistical analysis was done using one-way analysis of variance (ANOVA) with Šídák's multiple comparisons, ** $p < 0.01$.

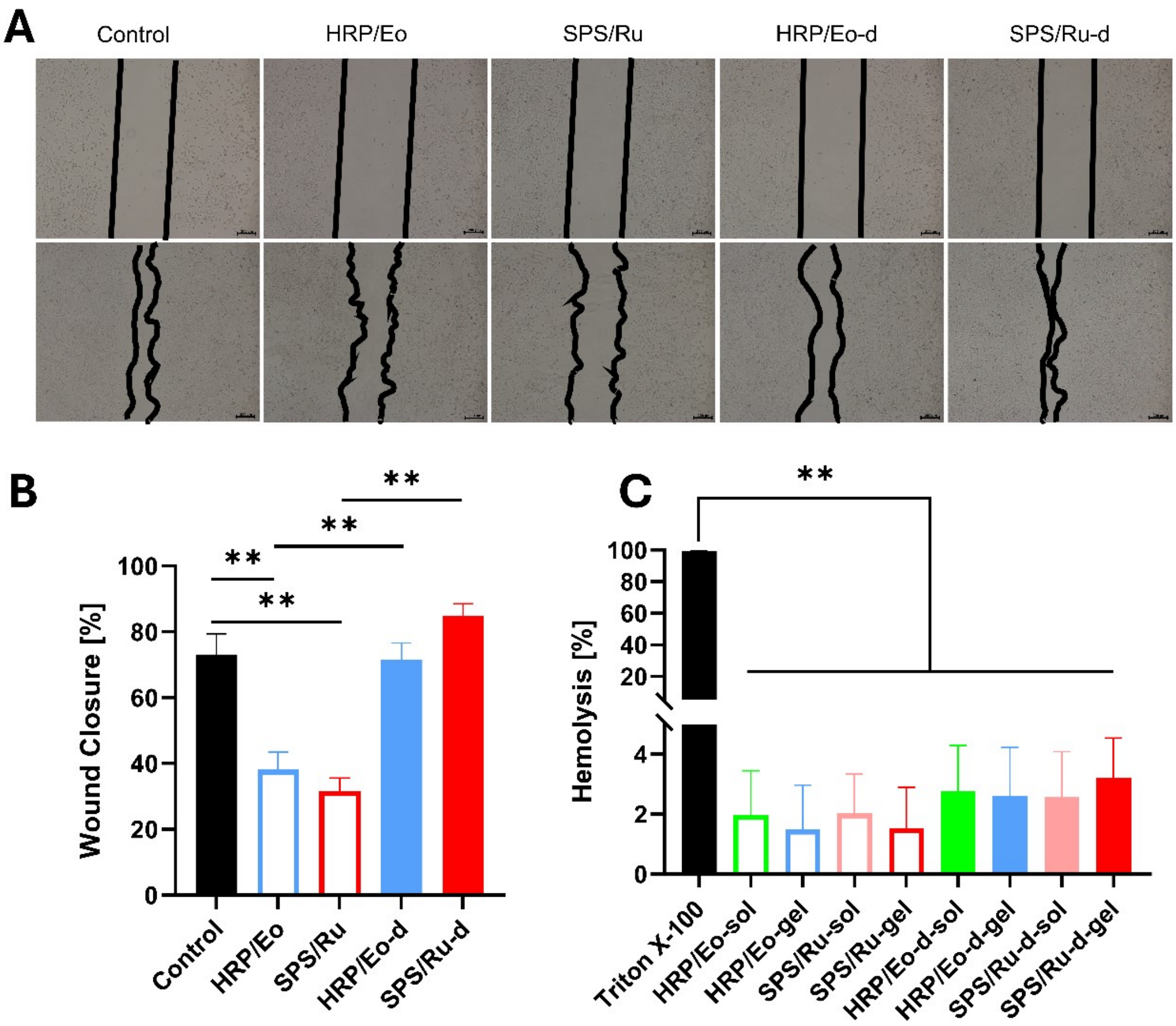

Fig. S4. Chondrocyte scratch assay and hemolysis evaluation. (A, B) Chondrocytes were treated with conditioned media derived from four hydrogel formulations, with basal medium serving as the control. (C) Hemolysis assay was performed using Triton X-100 as a positive control, evaluating eight groups including four hydrogel formulations before and after gelation. sol = solution from. Statistical analysis was done using one-way analysis of variance (ANOVA) with Šídák's multiple comparisons; *p* ** < 0.01.
